# Single-cell transcriptomic analysis reveals a systemic immune dysregulation in intravenous immunoglobulin non-responsive Kawasaki disease

**DOI:** 10.3389/fimmu.2025.1702290

**Published:** 2025-11-27

**Authors:** Chenhui Feng, Qirui Li, Minna Yang, Yeshi Chen, Mingming Zhang, Hongmao Wang, Xiaohui Li

**Affiliations:** 1 Department of Cardiovascular Medicine, Capital Center for Children’s Health, Capital Medical University, Beijing, China; 2 Capital Institute of Pediatrics, Peking University Teaching Hospital, Beijing, China; 3 Department of Cardiology, Beijing Children’s Hospital, Capital Medical University, National Centre for Children’s Health, Beijing, China; 4 Capital Institute of Pediatrics, Chinese Academy of Medical Sciences and Peking Union Medical College, Beijing, China; 5 Department of Pediatrics, Beijing Tsinghua Changgung Hospital, School of Clinical Medicine, Tsinghua Medicine, Tsinghua University, Beijing, China

**Keywords:** Kawasaki disease, single-cell transcriptomic analysis, IVIG non-responsive, inflammatory cytokine response, T/NK cell exhaustion, interferon

## Abstract

**Background:**

Intravenous immunoglobulin (IVIG) has been established as the first-line treatment for Kawasaki disease (KD). However, 10%–20% of patients are unresponsive, increasing their risk of coronary artery complications.

**Methods:**

To elucidate the pathogenesis of IVIG non-responsiveness, we performed single-cell transcriptomic profiling on 24 peripheral blood mononuclear cell (PBMC) samples from responsive and non-responsive KD patients before and after IVIG treatment. Finally, the expression of key cytokines was validated using public bulk RNA-seq data and enzyme-linked immunosorbent assay (ELISA).

**Results:**

Non-responders exhibited elevated inflammatory cells, lymphocyte dysfunction, and a stronger inflammatory cytokine response driven by the S100A12–TLR4–MYD88 axis, initiated by Mono_CD14_CD16 cells, which was closely associated with interferon activation. Despite T/NK-cell exhaustion, cytotoxic activity was preserved. All processes appeared to be closely associated with interferon activation. Disrupted Tfh–B-cell coordination was observed alongside plasmablast enrichment. Furthermore, monocytic myeloid-derived suppressor cells (MDSCs) suppressed T-cell function and promoted inflammation. The expression levels of S100A8/A9, S100A12, TNF, TNFSF8/10, and interferon-α were consistent with the transcriptomic data.

**Conclusions:**

Our findings reveal the immune landscape in IVIG non-responsive KD and suggest potential targets for alternative therapies.

## Introduction

Kawasaki disease (KD), also known as mucocutaneous lymph node syndrome, is a febrile illness characterized by acute onset and self-limiting progression. Its fundamental pathological manifestation is immune-mediated vasculitis. Coronary artery lesions (CALs) are the most severe complication and leading cause of acquired heart disease in children in developed countries (1, 2).

Intravenous immunoglobulin (IVIG) combined with oral aspirin is the standard first-line therapy for KD. The anti-inflammatory effects of IVIG are mediated by neutralization of infectious antigens or pathogenic autoantibodies, inhibiting the release of tumor necrosis factor (TNF)-α and inflammatory cytokines and modulating B-cell and T-cell function (3–5). However, approximately 10%–20% of KD patients are reportedly unresponsive to IVIG. Previous studies have indicated that elevated pro-inflammatory cytokines, including interleukin (IL)-6, IL-17, and TNF-α, are characteristic of IVIG non-responders (6). These cytokines are essential regulators of immune responses, inflammation, tissue repair, and metabolism. Notably, the hyperactivation of the NF-κβ signaling pathway, triggered by multiple mechanisms, may be a critical factor contributing to cytokine secretion and IVIG non-responsiveness (7, 8). Furthermore, increased expression of HLA-DR on CD4^+^ and CD8^+^ T cells, indicative of heightened T-cell activation during the acute phase of KD, may contribute to the development of IVIG non-responsiveness (9, 10). The therapeutic efficacy of IVIG relies on suppressing T-cell activation, which may be compromised when T-cell activation is excessively high (11). These findings provide valuable insights into immune cell dysfunction in IVIG non-responsive KD patients. Nonetheless, many questions remain unanswered about the mechanisms underlying these immune dysregulations, hindering the development and optimization of effective treatment strategies.

Single-cell RNA sequencing (scRNA-seq) enables the analysis of cellular heterogeneity in KD and the identification of distinct cell-type gene expression profiles (12–14). Wang et al. used scRNA-seq to reveal immune cell changes in KD before and after IVIG treatment, documenting the underlying mechanisms of IVIG (12). Chen et al. reported increased inflammatory cells (megakaryocytes and monocytes) during the acute stage of KD and an inflammatory cytokine storm in KD with CALs (13). Yang et al. reported that IVIG combined with methylprednisolone effectively downregulated monocyte-driven inflammatory pathways, enhanced NK-cell cytotoxicity, and mitigated NK-cell exhaustion by regulating receptor homeostasis (14). Zheng et al. analyzed scRNA-seq data from KD after IVIG treatment and revealed impaired CD8T_effector cell differentiation in IVIG non-responders with CALs (15). However, assessing IVIG non-responsiveness in KD before treatment remains challenging in clinical practice; few studies have reported the single-cell immune atlas of IVIG non-responders during the acute phase. This study aimed to explore transcriptomic changes in PBMCs from IVIG responders and non-responders before and after treatment via scRNA-seq analysis, offering deeper insights into the immune landscape of IVIG non-responsive KD.

## Methods

### Study design

The study was approved by the Ethics Committee of the Capital Institute of Pediatrics (SHERLL2023048). A total of 12 age- and gender-matched typical KD patients were enrolled in this study, consisting of 5 IVIG responsive and 7 IVIG non-responsive cases. PBMC samples were collected before and after IVIG treatment, yielding a total of 24 samples. Next, these samples were categorized into four groups based on the course of disease and response to IVIG treatment: IVIG responsive KD before treatment (BR, *n* = 5), IVIG responsive KD after treatment (AR, *n* = 5), IVIG non-responsive KD before treatment (BNR, *n* = 7), and IVIG non-responsive KD after treatment (ANR, *n* = 7). Participants were recruited from two centers between March 2022 and February 2024.

The diagnosis of KD was based on previous literature (2). Persistent fever >38 °C for more than 24 h or recurrent fever with KD symptoms following an afebrile interval within a week after the initial IVIG dose defines the criteria for IVIG non-responsive KD (16). All participants were confirmed to be negative for SARS-CoV-2 infection by PCR and serological testing and had no contact history. The blood sample collected before treatment was obtained following diagnosis of complete KD and before IVIG administration. One IVIG non-responder in our study received adjunctive steroid therapy. However, all post-treatment blood samples were collected 2–5 days after the initial IVIG infusion and before adjunctive steroid therapy. Informed consent was obtained from all participants and their guardians.

### Bulk RNA-sequencing data source

The bulk RNA-seq dataset used to validate our results was obtained from the GEO database (Accession ID: GSE18606) (17). The dataset included healthy controls (*n* = 9) as well as IVIG non-responsive (*n* = 8) and IVIG responsive KD patients at the acute and convalescent stages (*n* = 12). The IVIG non-responsive KD patients were aged between 0.33 and 4.92 years, with a male-to-female ratio of 5:3. IVIG responsive KD patients were aged between 0.33 and 9 years old, with a male-to-female ratio of 5:7.

### Single-cell RNA-sequencing and data analysis

For single-cell RNA library preparation and sequencing, 2 mL of whole blood was collected from each participant into EDTA vacutainers. PBMCs were isolated from fresh venous blood at baseline using a centrifuge and red blood cell lysis buffer (Miltenyi Biotec Bergisch Gladbach, Germany) and cryopreserved in liquid nitrogen. All PBMC samples were thawed, and cell viability >85% was confirmed with the Countstar cell viability kit. The MACS dead cell removal kit (Miltenyi Biotec) was then used to further enrich the PBMC suspension. Each sample required a minimum of 8,000 cells in a sufficient volume of PBMC suspension. Single-cell library construction was performed using the 10X Genomics Chromium Controller Instrument in conjunction with the Chromium Single Cell 5′ Library and Gel Bead Kit. Library quality was assessed by quantifying DNA with the Qubit High-Sensitivity DNA Assay (Thermo Fisher Scientific Waltham, MA, USA) and determining size distribution with the High-Sensitivity DNA Kit on an Agilent 2200 Bioanalyzer. Finally, sequencing was performed on an Illumina platform.

The scRNA-seq data were processed as previously described (13, 14, 18). In summary, a combined and filtered gene expression matrix of 24 samples was generated using kallisto/bustools (kb v0.24.4) and the ad.concat function in anndata (ad) (v0.7.6) (14). Subsequently, Scanpy (sc) (v1.9.2) was then employed to eliminate doublets/low-quality cells, normalize the library size to 10,000 reads per cell, and select a consensus set of the top 1,500 highly variable genes (HVGs) with high intercellular variability (14). Principal component analysis (PCA) was used for data integration, reducing the dimensionality to 20 PCA components. Batch effect correction was performed using the Harmony algorithm (19), and unsupervised clustering of the single-cell data was conducted using the Louvain algorithm (20).

### Cell clustering and annotations

Unsupervised clustering of cells was then computed by the sc.tl.louvain function from the Scanpy package in Python at different resolutions, based on cell neighborhood relations. There were two rounds of cell clustering in our study: 1) 10 major cell types were identified, including B cells, plasmablast, CD4^+^ T cells, CD8^+^ T cells, gamma delta T cells, NK cells, monocyte cells, myeloid dendritic cells (mDC), plasmacytoid dendritic cells (pDC), and megakaryocytes, in the first round (Louvain resolution = 2.0). 2) To further dissect the major immune subclusters present in B, CD4^+^/CD8^+^ T, monocyte, and NK cells, we performed the second round of analyses at the same resolution. These resulting subclusters, representing distinct immune cell lineages, were manually annotated based on the expression of canonical marker genes. The sc.tl.rank_genes_groups function was performed to identify signature genes specific to each cluster. Next, the specific signature genes were manually matched to lists of canonical cell marker genes (18, 21, 22) to annotate immune cell subclusters. A cluster was annotated if it expressed at least one signature marker gene. **Supplementary Table 1** lists the canonical marker genes and the highly expressed signature genes specific to each cluster.

### Identifying changes in immune cell proportion

The proportions of every immune cell type/subtype under different groups were calculated, and statistical significance was assessed using Student’s *t*-test. Additionally, multivariate analysis of variance (ANOVA) was performed to elucidate the effects of different disease conditions and treatment responsiveness, as well as their potential interactions on the proportions of each cell type/subtype. Subsequently, the ratio of observed to expected cell counts (*R*_O/E_) was calculated to determine the specific enrichment for each type/subtype.

### Cell-state score of immune cell subtypes

Following cluster annotation, we compared the physiological activity or overall activation level of cell clusters using defined gene sets. Based on a previous study (13), B-cell activation, antigen presentation, the inflammatory response, and pro-inflammatory cytokines were collected. The gene sets associated with exhaustion state, cytotoxic state, IFN response, platelet activation, and platelet aggregation were obtained from previous reports (14, 23) (**Supplementary Table 2**). By averaging expression across predefined gene sets relative to the reference genes, we computed cell-state scores using the Scanpy sc.tl.score_genes function. The cell-state scores were compared between groups by means of the Mann–Whitney rank test (two-tailed, *p* < 0.01, corrected using the Benjamini–Hochberg procedure) statistically.

### Partition-based graph abstraction analysis

Partition-based graph abstraction (PAGA) was employed to visualize the developmental trajectories of cell subclusters within a graph-based framework in Scanpy (v1.5.1) with default parameters.

### Statistics

All statistical analyses, visualizations, and methodologies described in our study were implemented in Python. Comprehensive descriptions of the statistical methods can be found in the main text, figure legends, or within this section. In all figures, significant marks are defined as follows: ns: *p* > 0.05; *p < 0.05; **p < 0.01; ***p < 0.001; ****p < 0.0001.

## Results

### Single-cell transcriptional profiling of PBMCs from individuals with KD

**Figure 1:**
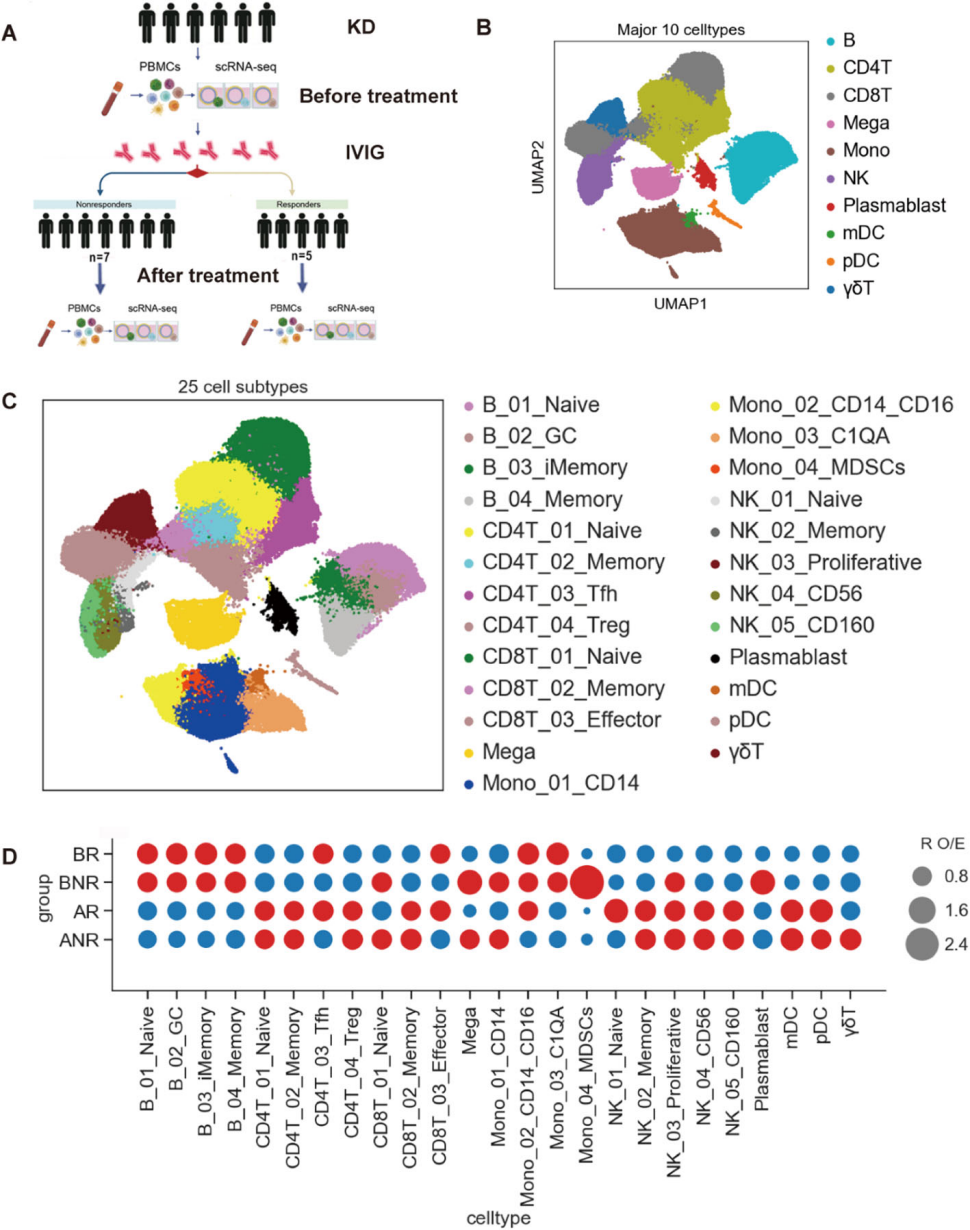
Study design and overview of results from the single-cell transcriptomics of PBMCs. **(A)** Overall study design. Twenty-four blood samples from 12 KD patients, 5 IVIG responders, and 7 IVIG non-responders, both before and after IVIG treatment. **(B)** The clustering results of the 10 major cell types (right panel) across the 24 samples (left panel). Each dot represents a single cell, colored by cell type. **(C)** UMAP projection displaying 25 identified cell subtypes across the 24 samples. Each dot represents an individual cell, with colors indicating the respective cell subtypes. **(D)** Dot plot depicting the 25 cell subtypes’ preference of disease state and IVIG treatment response as calculated using *R*_O/E_. The color presents the state of enrichment or depletion of each cluster. The size of the dot presents the magnitude of enrichment or depletion of each cluster. KD, Kawasaki disease; IVIG, intravenous immunoglobulin; BR, IVIG responders before IVIG treatment; AR, IVIG responders after IVIG treatment; BNR, IVIG non-responders before IVIG treatment; ANR, IVIG non-responders after IVIG treatment.

Twenty-four PBMC samples underwent scRNA-seq analysis to explore the immunological profile underlying IVIG non-responsive KD (**Figure 1A**; **Table 1**). Clinical and laboratory examination data of these patients are detailed in **Supplementary Table 3**.

**Table 1:** Demographics of the patient cohorts undergoing single-cell analysis of peripheral blood.

Group	Patients and comments		Sex	Age (months)	Time from KD onset to diagnosis (days)	Time from KD onset to blood draw (days)	Dose of IVIG (g/kg)	Time from IVIG treatment to the second blood draw (days)
IVIG responsive KD patients	P1	BR1	Male	11	4	4	2g/kg	4
		AR1						
	P2	BR2	Male	15	6	6	2g/kg	4
		AR2						
	P3	BR3	Female	6	4	4	2g/kg	4
		AR3						
	P4	BR4	Male	22	5	5	2g/kg	4
		AR4						
	P5	BR5	Female	10	6	6	2g/kg	3
		AR5						
IVIG non-responsive KD patients	P1	BNR1	Female	48	7	7	2g/kg	5
		ANR1						
	P2	BNR2	Female	45	4	4	2g/kg	3
		ANR2						
	P3	BNR3	Male	14	7	7	2g/kg	3
		ANR3						
	P4	BNR4	Female	24	4	4	2g/kg	5
		ANR4						
	P5	BNR5	Male	36	7	7	2g/kg	2
		ANR5						
	P6	BNR6	Male	73	9	9	2g/kg	5
		ANR6						
	P7	BNR7	Male	13	4	4	2g/kg	4
		ANR7						

Following quality control (QC) (**Supplementary Material**), 155,949 high-quality cells underwent sequencing (**Supplementary Figures 1A, B**). After correcting for mitochondrial read counts and read depth and applying PCA to integrate data into unbatched and comparable datasets (**Supplementary Figures 1A, B**), 31,215 (20.01%), 35,092 (22.51%), 39,953 (25.62%), and 49,689 (31.86%) cells were obtained from the BR, AR, BNR, and ANR groups. Based on this, we identified 10 major cell types and 25 subtypes using classic marker gene expression and uniform manifold approximation and projection (UMAP) clustering (**Figures 1B, C**; **Supplementary Figures 1C, D, 2****;****Supplementary Table 1**). Most of these cell types were presented across all groups, indicating a shared immune profile.

**Supplementary Figure 3** illustrates the relative proportions of the major immune cell types across different groups. Before treatment, all patients exhibited reduced lymphocyte proportions (CD4^+^ T cells, CD8^+^ T cells, γδT cells, and NK cells) and increased proportions of B cells and monocytes, consistent with previous findings (13). Notably, compared to IVIG responders, IVIG non-responders showed an increased trend of inflammatory cells (monocytes and megakaryocytes) and plasmablast but a decreased trend of B-cell and CD4^+^ T-cell proportions. In addition, an opposite change trend was observed in plasmablast between the IVIG responsive and IVIG non-responsive KD patients after IVIG treatment.

Each cell subset displayed different sample origins and enrichment in IVIG responders (**Figure 1D**; **Supplementary Figure 4A**). To analyze the unique immune profiles of different groups, we investigated the immune cell composition in each individual (**Figure 1D**; **Supplementary Figure 4**). In IVIG non-responsive KD, inflammatory cells (Mono_CD14, Mono_MDSCs, and megakaryocytes) were significantly enriched. Among T and NK cells, naive or proliferative cells (CD8_Naive and NK_Pro) were enriched, while effector T-cell subsets (CD4_Tfh, CD4_Treg and CD8_Effector) showed a decreasing trend (**Figure 1D**; **Supplementary Figure 4**). This pattern suggests that IVIG non-responsive KD may exhibit a more pronounced inflammatory response, accompanied by potential dysregulation of lymphocyte function (6, 11, 24). Taken together, these results indicated that these cell types may be associated with IVIG non-responsiveness. Thus, we speculated that IVIG non-responsive KD may exhibit an altered circulating immune profile, particularly in pro-inflammatory cells and the adaptive immune system.

### Heightened inflammatory cytokine response in monocytes of IVIG non-responsive KD

**Figure 2:**
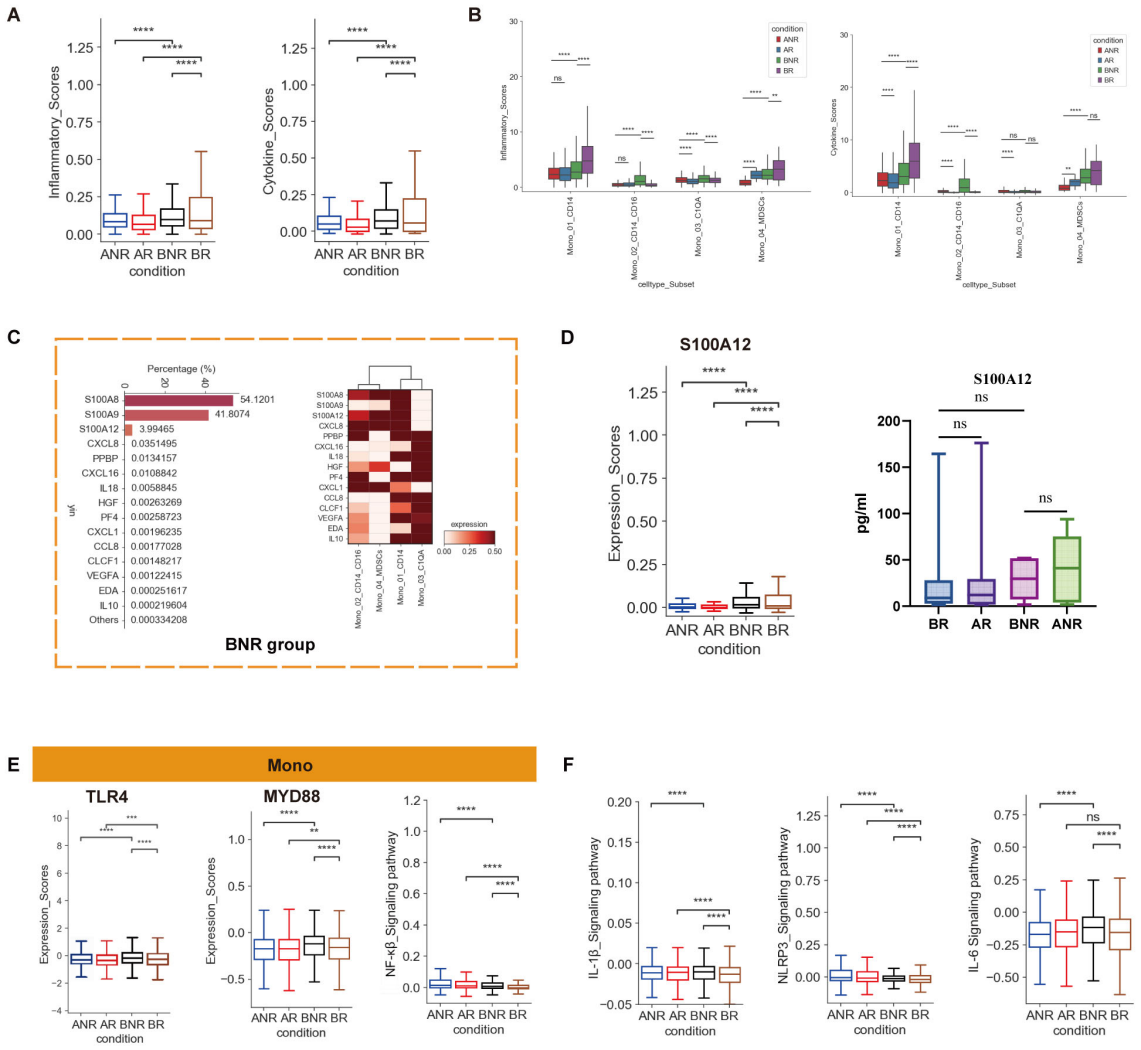
The high inflammatory cytokine response in monocytes of IVIG non-responders. **(A)** Box plots showing the inflammatory scores and cytokine scores in total monocytes between different groups. **(B)** Box plots depicting the inflammatory scores and cytokine scores in different monocyte subsets across different groups. **(C)** Bar chart depicting the relative contribution of the top 15 cytokines in IVIG non-responders before treatment and the heatmap showing the expression of these 15 cytokines within each monocyte subtype. **(D)** Box plots showing the expression levels of S100A12 by scRNA-seq and ELISA. **(E)** Box plots illustrating the expression of TLR4, MYD88, and NF-κβ signaling pathway in total monocytes. **(F)** Box plot showing the signaling pathway scores associated with IVIG non-response in monocytes between different groups. Significant differences were determined by two-sided Student’s *t*-test with Bonferroni correction (*p < 0.05, **p < 0.01, ***p < 0.001, ****p < 0.0001, ns *p* > 0.05).

Monocytes have been confirmed as the primary source of inflammatory response in KD (13, 14). In our cohort, all monocyte clusters were enriched among IVIG non-responders during the acute phase. Classical monocytes (Mono_CD14), intermediate monocytes (Mono_CD14_CD16), and Mono_MDSCs cell subsets were most prominent (**Figure 1D**, **Supplementary Figure 4**), consistent with previous findings (12, 25). Then, using previously reported pro-inflammatory response and cytokine gene data (14) (**Supplementary Table 2**), we evaluated the inflammatory and cytokine scores of monocytes across groups. Increased tendency of inflammatory and cytokine scores was observed in IVIG non-responders (**Figure 2A**), suggesting a strong inflammatory cytokine response. These scores were then utilized to assess the contributions of each monocyte subtype to inflammatory cytokine response under four conditions. Before treatment, Mono_CD14 and Mono_MSDCs exhibited high inflammatory cytokine scores in IVIG responders, whereas Mono_CD14_CD16 and Mono_C1QA scores were elevated in IVIG non-responders (**Figure 2B**). Thus, high inflammatory cytokine response in monocytes, particularly in the Mono_CD14_CD16 and Mono_C1QA clusters, might be the key factor for IVIG non-responsive KD.

Moreover, most cytokines secreted by monocytes, including *TNF*, *TNFSF4/13*, *IL6*, *CXCL9/16*, and *CCL28*, displayed contrasting expression trends in IVIG responsive and non-responsive KD after IVIG treatment, suggesting their potential as therapeutic targets (**Supplementary Figure 5A**). Specific cytokines associated with the inflammatory response differed between IVIG responsive and non-responsive KD. In this respect, 15 cytokines, including *S100A8/A9/A12* and *CXCL1/8/16*, constituted over 99% of cytokine scores in IVIG non-responders before treatment (**Figure 2C**). The combined score of *S100A8/A9/A12* accounted for more than 99% of the total score. Similarly, the expression level of *S100A8/A12* in monocytes was also elevated in IVIG non-responders, as confirmed by ELISA (**Figure 2D**, **Supplementary Figure 5B**). Among them, *S100A12* exhibited the most pronounced increase. Interestingly, our analysis of the main subsets producing these cytokines revealed that, in IVIG non-responders, the Mono_CD14_CD16 subset is a distinct population secreting *S100A8/A12*. Our previous study has identified the Mono_CD14_CD16 subset as the major source of inflammatory response in KD with CALs (13), suggesting that cytokines from this subset are crucial for the treatment response and prognosis of KD. *S100A12* can bind to TLR4, activating the signaling mediator MYD88, which ultimately leads to NF-κB activation (**Figure 2E**) and secretion of pro-inflammatory cytokines such as TNF-α, IL-1β, and IL-6 (23). These processes may be closely associated with IVIG non-response. Conversely, Mono_CD14, Mono_CD14_MDSCs, and Mono_C1QA were the main cytokine producers in IVIG responders (**Supplementary Figures 5C–E**). ELISA within our cohort also demonstrated high expression of TNF families members [TNF-α, TNFSF8 (also known as CD30L), and TNFSF10 (also known as TRAIL)] in IVIG non-responsive KD, corresponding with our scRNA-seq results (**Supplementary Figure 5F**). This highlights the importance of inflammatory cell subsets (Mono_CD14_CD16 subset) and molecules (S100A12, TNF, TNFSF8, and TNFSF10) in the occurrence of IVIG non-responsive KD. Furthermore, some inflammatory signaling pathways, which have been reported to be associated with IVIG non-responsive KD (7, 26, 27), were also upregulated in monocytes of IVIG non-responsive KD (**Figure 2F**). Collectively, these findings highlight the strong inflammatory cytokine response in monocytes that contributes to the development of IVIG non-responsiveness.

### Naive and highly exhausted CD8 T cells in IVIG non-responsive KD

**Figure 3:**
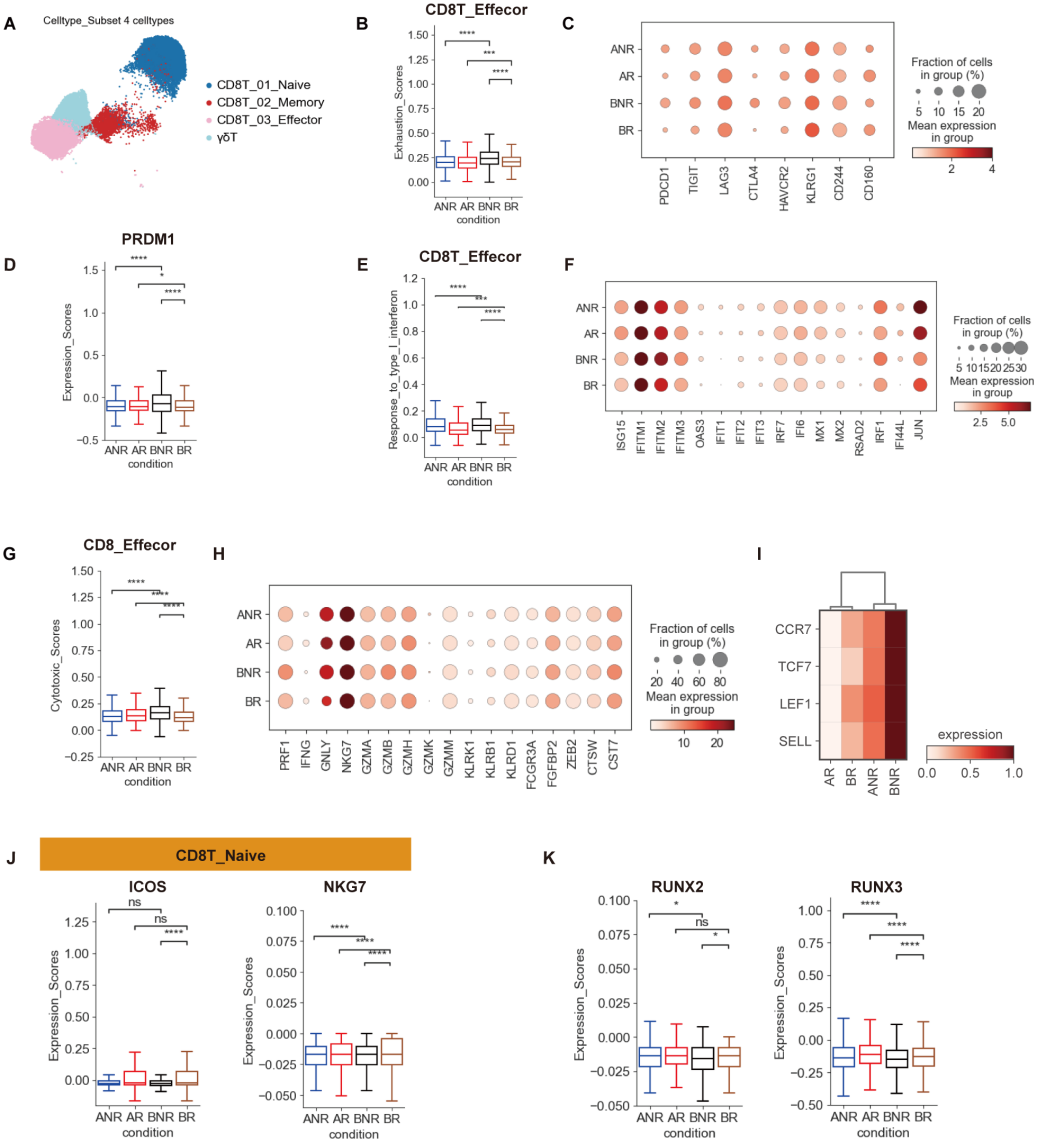
Naive and high exhausted CD8T cells in IVIG non-responsive KD. **(A)** The UMAP clustering result of CD8^+^ T subsets. Each point represents one single cell, colored according to cell type. **(B)** Box plots showing the exhaustion scores in CD8_Effector cells between different groups. **(C)** Dot plot demonstrating the expression of selected exhaustion genes in CD8_Effector cell subtype across different groups. **(D)** Box plots illustrating the expression of PRDM1 in CD8_Effector cells. **(E)** Box plots showing the scores of the IFN-I pathway in CD8_Effector cells between different groups. **(F)** Dot plot demonstrating the expression of IFN-I-related genes in the CD8_Effector cell subtype across different groups. **(G)** Box plots showing the cytotoxicity scores in CD8_Effector cells between different groups. **(H)** Dot plot demonstrated the expression of selected cytotoxicity genes in the CD8_Effector cell subtype across different groups. **(I)** Heatmap showing the relative expression of early T-cell development-related genes (CCR7, TCF7, LEF1, and SELL) in CD8 T cells. **(J)** Box plots showing ICOS and NKG7 expression in CD8_Naive cells between different groups. **(K)** The expression of RUNX2 and RUNX3 genes in CD8^+^ T cells across different groups. *p < 0.05; ***p < 0.001; ****p < 0.0001.

Next, four distinct CD8^+^ T-cell subtypes were identified: CD8T_01_Naive, CD8T_02_Memory, CD8T_03_Effector, and γδT cells (**Figure 3A**). Preference analysis across the four groups revealed an enrichment of the CD8T_Naive subtype in IVIG non-responders, while the CD8T_Effector subset was enriched in IVIG responders and depleted in IVIG non-responders (**Figure 1D**). Additionally, IVIG non-responders showed an upward trend in the proportion of γδT cells, potentially reflecting enhanced innate immune activation (**Supplementary Figure 3C**). The enrichment of CD8T_Naive cells and the reduction of the CD8T_Effector subset suggest impaired CD8^+^ T-cell-mediated adaptive immune responses in IVIG non-responders. Collectively, these findings underscore distinct alterations in CD8^+^ T-cell subsets between IVIG responders and non-responders in KD patients.

The CD8T_Effector and γδT cell subsets displayed a pronounced exhaustion phenotype (**Figure 3B**; **Supplementary Figures 6A, B**), suggesting that dysfunction of CD8^+^ T cells may be a key contributor to IVIG resistance. In comparison to IVIG responders, CD8T_Effector and γδT cells from IVIG non-responders exhibited increased expression of multiple exhaustion-related genes, including *PDCD1*, *TIGIT*, *CTLA4*, and *HAVCR2* (**Figure 3C**; **Supplementary Figure 6C**). It has been reported that PDCD1 interacts with PD-L1 or PD-L2, whereas HAVCR2 (Tim-3) binds to galectin-9. These interactions recruit the tyrosine-protein phosphatases SHP1 and/or SHP2 via intracellular signaling domains, including the immunoreceptor tyrosine-based switch motif (ITSM) and the immunoreceptor tyrosine-based inhibitory motif (ITIM) (23). These interactions reduce cellular proliferation and cytokine secretion. Moreover, elevated *PRDM1* expression was associated with functionally exhausted cells, marked by diminished polyfunctionality and increased inhibitory receptor expression (23). Correspondingly, CD8_Effector cells exhibited an upregulated expression of *PRDM1* in IVIG non-responders (**Figure 3D**). CD8^+^ T-cell exhaustion is strongly linked to sustained type I interferon (IFN-I) signaling (28). In the present study, significant enrichment of IFN-I signaling pathway and IFN-I-related genes was observed in exhausted CD8_Effector and γδT cells, indicating a direct association between T-cell exhaustion and sustained IFN signaling (**Figures 3E, F**; **Supplementary Figures 6D, E**). Together, these results suggest that the exhaustion observed in the CD8^+^ T subsets of IVIG non-responders is likely driven by sustained type I IFN signaling.

Although showing exhaustion features, the exhausted CD8^+^ T-cell subsets in IVIG non-responsive KD exhibited high cytotoxicity scores and increased expression of cytotoxicity-associated genes, including *PRF1*, *GNLY*, *NKG7*, *GZMB*, and *GZMK*, suggesting functional heterogeneity within these exhausted subsets (**Figures 3G, H**; **Supplementary Figures 6F–H**). These findings align with previous studies (21, 28), indicating that while exhausted CD8^+^ T cells may have reduced proliferative and cytokine-producing abilities, their cytotoxic potential largely remains intact. While granule-mediated cytolytic functions of T cells (e.g., granzyme, granulysin, and perforin) are crucial for eliminating target cells, their overexpression may induce immunopathology by triggering inflammatory responses. Thus, it can be inferred that elevated levels of cytolytic proteins in T cells (particular CD8T_Effector) are linked to IVIG non-responsiveness, despite their small proportion.

To further characterize CD8^+^ T cells in the patient groups, we performed DEG analysis. Naive T-cell-associated genes (*CCR7*, *TCF7*, *LEF1*, *SELL*) were upregulated in IVIG non-responders (**Figure 3I**). However, genes promoting CD8T_Naive activation, including *ICOS* and *NKG7*, showed a downward trend in IVIG non-responders, indicating that their CD8^+^ T cells remain naive (**Figure 3J**). Consistently, genes regulating T-cell proliferation, differentiation, and function (*RUNX2*, *RUNX3*, *BATF*, *TBX21*, *EOMES*) were downregulated (**Figure 3K**; **Supplementary Figure 6I**). DEGs further revealed enrichment of IFN-I-related, HLA-I antigen presentation, and exhaustion-associated genes in IVIG non-responders, consistent with GO analysis, suggesting their role in IVIG resistance (**Supplementary Figures 6J, K**). In contrast, HLA class II genes (*HLA-DPA1*, *HLA-DQA1*, *HLA-DRB1*, *HLA-DRB5*) were downregulated in CD8^+^ T cells of IVIG non-responders, indicating a state of immune paralysis in these cells (**Supplementary Figure 6J**).

### Highly exhausted NK cells in IVIG non-responsive KD

**Figure 4:**
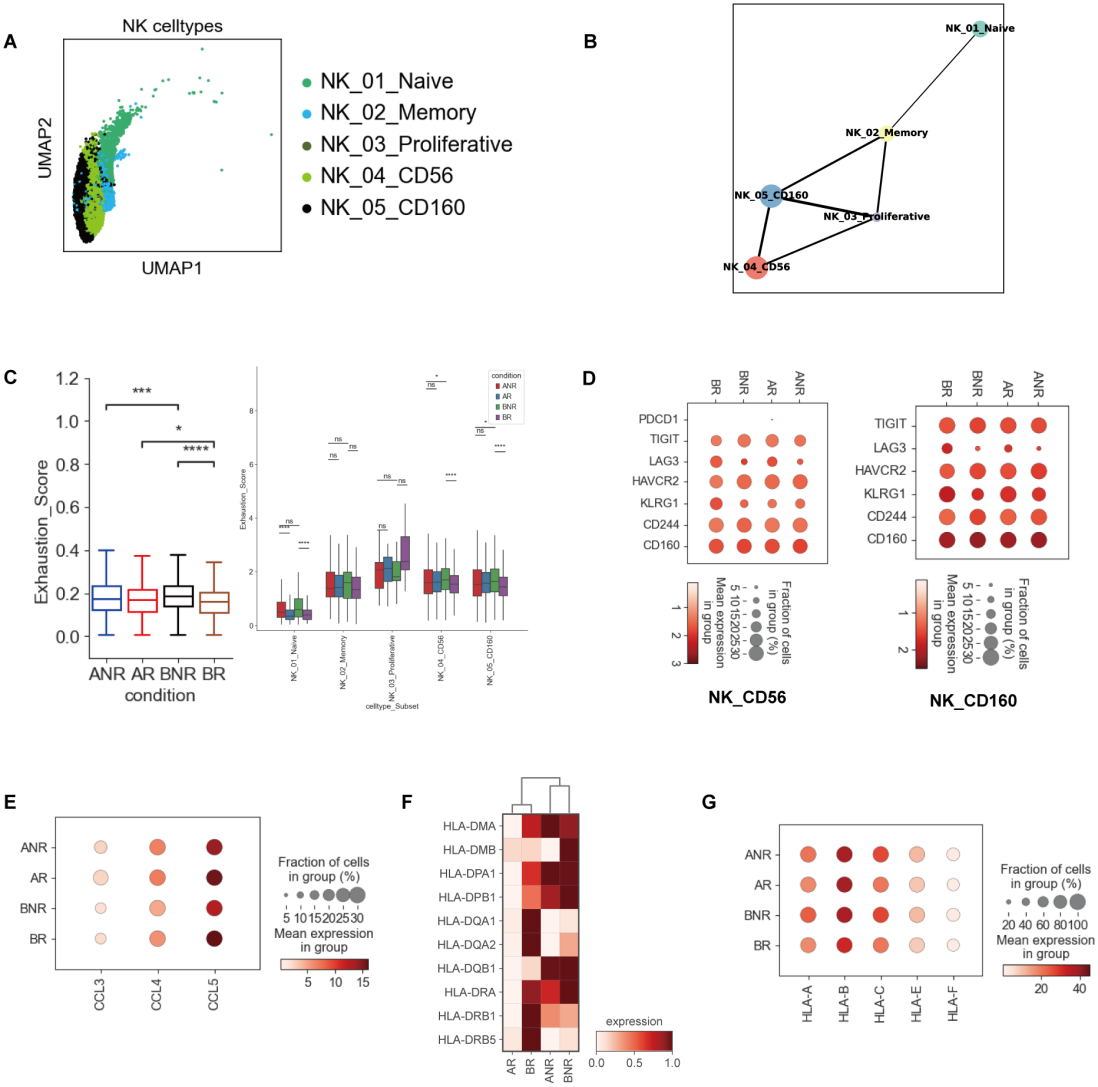
Highly exhausted NK cells in IVIG non-responsive KD. **(A)** The UMAP clustering result of NK subsets. Each point represents one single cell, colored according to cell type. **(B)** PAGA analysis of NK-cell pseudo-time: the associated cell type is shown. **(C)** Box plots of exhaustion scores in total NK cells (left) and each NK-cell subset (right). **(D)** Dot plots showing the relative expression of selected exhaustion markers in NK_CD56 and NK_CD160 subsets. **(E)** Dot plot depicting the expression of NK-produced cytokines in NK_CD56 cells. **(F)** Heatmap showing the relative expression of HLA class II molecules in NK cells. **(G)** Dot plot showing the expression of HLA-I genes in NK cells across each group. *p < 0.05; ***p < 0.001; ****p < 0.0001.

We identified six NK-cell subsets: NK_01_Naive, NK_02_Memory, NK_03_Proliferative, NK_04_CD56, and NK_05_CD160 (**Figure 4A**), each exhibiting distinct associations with clinical response. NK_Naive and NK_Memory subsets showed a declining trend in IVIG non-responders (**Figure 1D**; **Supplementary Figure 4**). We next applied PAGA to examine global connectivity and potential cellular trajectories underlying NK-cell state transitions. Several nodes exhibited strong intercluster connectivity, suggesting potential cross-differentiation bridges among NK-cell subsets (**Figure 4B**). The NK_Memory subset appeared to serve as an intermediate state, linking naive NK cells to all other subsets (NK_Pro, NK_CD56, and NK_CD160). Consistently, NK_Memory cells from non-responders expressed higher levels of activation-associated genes (*KLRK1*, *KLRC1*, *KLRD1*, *KLRF1*) (**Supplementary Figure 7**). The proliferative NK subset (NK_Pro), characterized by high expression of *MKI67* and *TYMS*, was enriched in IVIG non-responders. NK_Pro cells primarily originated from the NK_Memory subset and showed strong connectivity with NK_CD56 and NK_CD160 clusters (**Figure 4B**). However, NK_Pro cells showed downregulation of activation-related receptors (*KLRK1*, *KLRC1*, *KLRC2*, *KLRC3*), potentially explaining the lack of coordinated expansion in NK_CD56 and NK_CD160 subsets (**Supplementary Figure 7**). Therefore, targeting NK_Pro cells may offer therapeutic potential.

IFN-I-related pathways were also upregulated in NK cells (**Supplementary Figures 8, 9A**). A previous study has shown that the IFN-I signaling pathway can enhance NK-cell cytotoxicity and IFN-γ production, promoting the survival and accumulation of proliferating NK cells (29). Our findings also confirmed the enrichment of genes associated with type I IFN signaling (*ISG15*, *IFIT1*, *IFITN3*, etc.), cell proliferation (*MKI67*), and cytotoxic effector molecules in NK cells from IVIG non-responders (**Supplementary Figures 9B, C**). These findings further explain the expansion of the NK_Pro subset in IVIG non-responders.

We next analyzed the exhaustion score and gene expression in NK cells among different groups. Total NK cells and their subpopulations exhibited high exhaustion scores in IVIG non-responders (**Figure 4D**). Then, we compared the exhaustion marker expression between different groups. Notably, the expression of *HAVCR2*, *CD244*, and *TIGIT* was raised in NK_CD56 and NK_CD160 in IVIG non-responders (**Figure 4E**). Immune cell exhaustion has been reported to reduce cytokine production while preserving cytotoxic function (13). Consistent with this, cytokine-encoding genes (*CCL3*, *CCL4*, and *CCL5*) were downregulated in NK_CD56 (**Figure 4F**). NK_CD16 exhibited comparable patterns (**Supplementary Figure 9D**). Taken together, these findings revealed that highly exhausted NK cells may contribute to IVIG non-responsiveness.

Furthermore, genes encoding HLA class II molecules were upregulated in IVIG non-responders (**Figure 4G**). Similarly, high expression of HLA class I genes, including canonical (*HLA-A/B/C*) and non-canonical (*HLA-E/F*) HLA-I molecules, were observed in IVIG non-responders (**Figure 4H**). These findings may indicate enhanced interactions between NK cells and other immune cells (e.g., elevated crosstalk between NK and DCs cells) (30).

### Dysregulated humoral immune and Treg response in IVIG non-responsive KD

**Figure 5:**
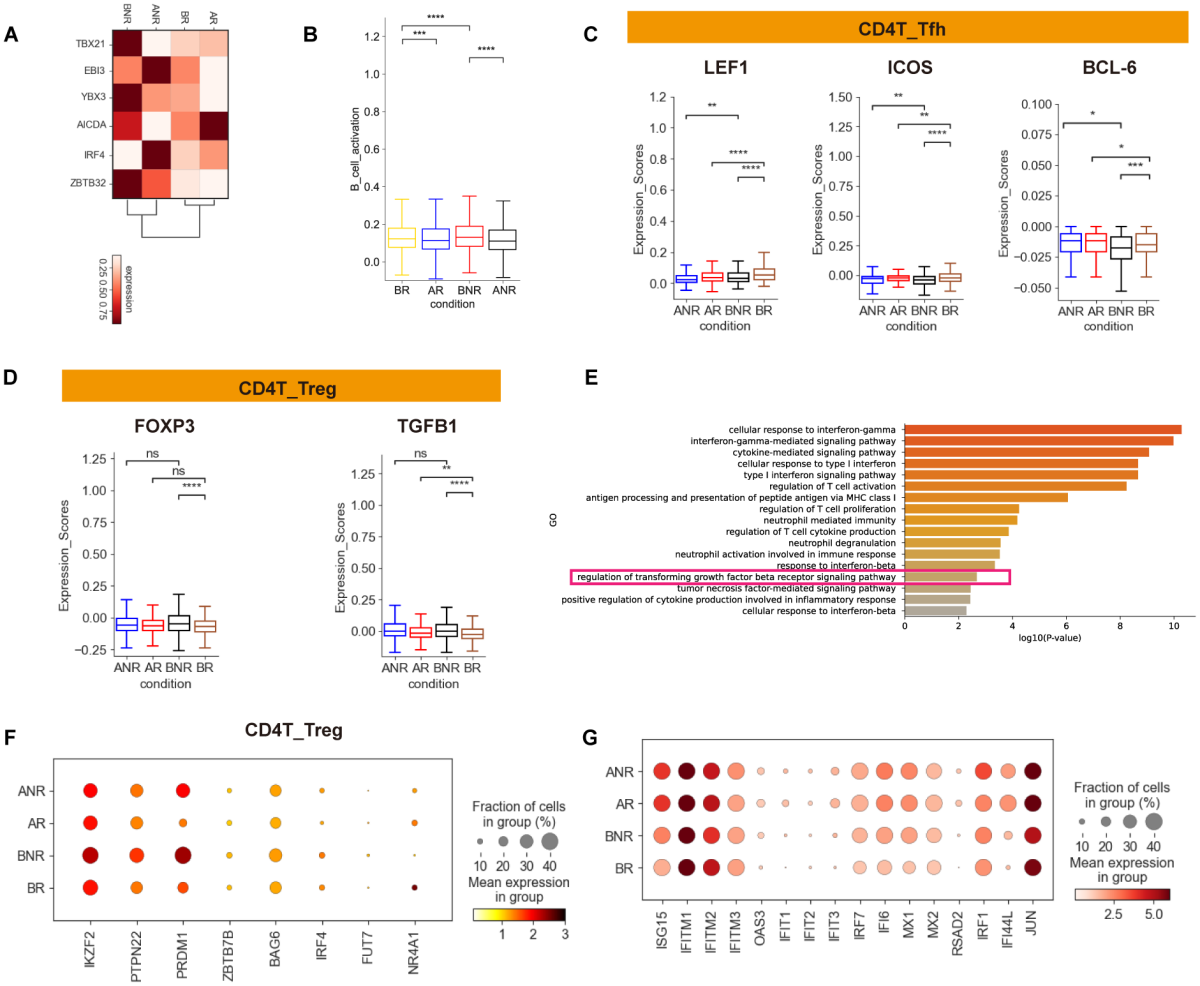
Dysregulated humoral immune and Treg response in IVIG non-responsive KD. **(A)** Heatmap showing the gene expression of B memory activation within different groups in the B_Memory cell subset. **(B)** Box plots exhibited the B-cell activation scores in different groups. **(C)** Box plots exhibiting LEF1, ICOS, and BCL-6 expression in CD4_Tfh cells between different groups. Significant differences were determined with a two-sided Student’s *t*-test with Bonferroni correction. Standard error (SE) and median are shown. **(D)** Box plots showing FOXP3 and TGFB1 expression in CD4_Treg cells between different groups. **(E)** GO enrichment analysis of upregulated DEGs in IVIG non-responders compared with IVIG responders before treatment in CD4^+^ T cells. DEGs refer to genes with a Wilcoxon-adjusted *p*-value <0.05. **(F)** Dot plot depicting the expression of selected exhaustion genes in CD4_Treg cells. **(G)** Dot plot depicting the expression of type I IFN-related genes in CD4_Treg cells between different groups. *p < 0.05; **p < 0.01; ***p < 0.001; ****p < 0.0001.

Compared with IVIG responders, IVIG non-responders exhibited a declining trend in the proportion of B cells and CD4^+^ T cells, indicating dysregulated humoral immunity in these patients (**Supplementary Figure 3**). Notably, IVIG non-responders demonstrated an enrichment of B_memory (memory B cells) and plasmablast subsets before treatment. Transcription factors associated with memory B-cell activation, including *EBI3* and *AICDA*, were upregulated. Furthermore, regulators controlling memory B-cell activation, such as *TBX3*, *TBX21*, and *ZBTB32*, were also elevated in IVIG non-responders (**Figure 5A**). Consistently, overall B-cell activation was enhanced in IVIG non-responsive KD (**Figure 5B**). The B_Memory cluster has previously been identified as the major source of plasmablasts. Elevated expression of *CD38*, *MZB1*, M*KI67*, *XBP1*, and *PRDM1* was observed in plasmablasts, confirming their identity as highly proliferative cycling plasma cells. Plasmablasts also displayed increased expression of immunoglobulin constant region genes (*IgM*, *IgG1–IgG4*, *IgA1*, and *IgA2*), emphasizing their role in secreting antigen-specific antibodies (**Supplementary Figure 2**, **Supplementary Table 1**). These findings suggest that IVIG non-responders may exhibit elevated serum antibody levels. Together, early activation of B cells (particularly in memory B cells) together with plasmablast expansion may represent a key immunological feature distinguishing IVIG non-responders.

Expansion of Tfh cells is typically accompanied by an increase in plasmablasts and the upregulation of B-cell activation-related genes, indicating an effective humoral immune response (31). Notably, the coordinated Tfh–B-cell axis is important for generating high-affinity antibodies and establishing long-term immunity, and disruption in severe cases may contribute to disease progression (21). Interestingly, we found a reduction trend in CD4_Tfh in IVIG non-responders (**Supplementary Figure 4C**). The activation-related gene expression (*LEF1*, *ICOS*, and *BCL6*) of the CD4_Tfh cluster as also reduced in IVIG non-responders (**Figure 5C**), contrasting with the results in plasmablasts. These results indicated a disrupted coordination between T- and B-cell responses in IVIG non-responders.

Furthermore, transcription factors *FOXP3* and *TGFB1* were upregulated in IVIG non-responders (**Figure 5D**), which are essential for the development and regulation of CD4_Treg cells. GO term (“regulation of transforming growth factor beta receptor signaling pathway”) was also enriched (**Figure 5E**). Treg cells produce TGF-β, suppressing CD4^+^ T-cell responses, inhibiting cytokine production, and downregulating effector immune responses (32). Thus, high expression of TGF‐β indicated that immune regulation by Treg cells may also be related to immune tolerance in IVIG non-responders (33, 34). Moreover, the CD4_Treg subpopulation showed elevated expression of exhaustion markers, including *IKZF2*, *PTPN22*, and *PRDM1* (**Figure 5F**). Exhausted Treg cells may result in a loss of regulatory function, leading to exacerbated immune-inflammatory responses. Interestingly, IFN-I response was also observed in CD4_Treg cells from IVIG non-responders (**Figure 5G**), similar to the results in other immune cells (**Supplementary Figure 8**). Accordingly, targeting the CD4_Treg subset could be a potential therapeutic strategy.

### Dysregulation of myeloid cell function in IVIG non-responsive KD

**Figure 6:**
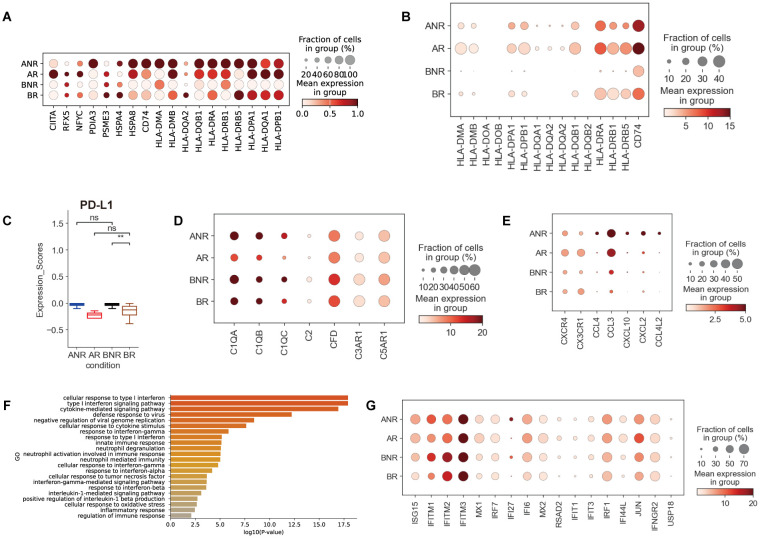
Dysregulation of myeloid cell function in IVIG non-responsive KD. **(A)** Dot plot illustrating the expression of antigen presentation-associated genes in mDCs. **(B)** Dot plot depicting the expression of HLA class II genes in Mono_MDSCs cells between different groups. **(C)** Box plots showing PD-L1 expression in Mono_MDSCs cells between different groups. **(D)** Dot plot showing the expression of complement-related genes in Mono_C1QA cells across different groups. **(E)** Comparison of genes associated with leukocyte chemotaxis in the monocyte subset across different groups. **(F)** GO enrichment analysis of DEGs in IVIG non-responders compared with IVIG responders before treatment in monocyte cells. DEGs refer to genes with a Wilcoxon-adjusted *p-*value <0.05. **(G)** Dot plot showing the expression of type I interferon genes in monocytes.

Dendritic cells (DCs), particularly classical DCs (mDCs), specialize in antigen processing and play a central role in initiating innate immunity and triggering robust adaptive immune responses. Taking this into consideration, we investigated the antigen-presenting and phagocytosis capacities of mDCs in IVIG non-responsive KD. Effective antigen presentation is predominantly mediated by major HLA class II molecules. Compared with IVIG responders, IVIG non-responders exhibited reduced expression of HLA class II molecules (**Figure 6A**). Beyond HLA class II molecules, key regulators of mDC-mediated antigen presentation, including *CIITA*, *NFYC*, *HSPA4*, and *HSPA8*, were also downregulated in IVIG non-responders (**Figure 6A**). Furthermore, mDCs displayed altered phagocytosis function, reflected by reduced expression of phagocytosis-related genes such as *CDC42*, *WASF2*, *PIK3CG*, *LYN*, and *PTPRC* in IVIG non-responders (**Supplementary Figures 10A, B**). Collectively, these findings indicated impaired antigen presentation and phagocytosis capacity in IVIG non-responders before treatment, likely disrupting normal immune response.

Among myeloid cells, a monocyte cluster (Mono_MDSCs) showed a strong association with IVIG non-responders. For a more detailed analysis, Mono_MDSCs were characterized by higher expression of inflammatory molecules (e.g., *S100A8/A12*) and lower expression of HLA-II gene (e.g., *HLA-DRB1*, *HLA-DPB1*, *HLA-DMA*) molecules, compared to other monocytes (**Supplementary Figure 10C**). Low HLA‐DR expression is a well-established marker of monocyte dysfunction, resulting in a diminished immune response (35, 36), indicating that the Mono_MDSCs cluster is the dysfunctional monocytes. We also found the declined expression of HLA-II genes in IVIG non-responders (**Figure 6B**), which further supports the dysfunctional monocytes of this cluster in IVIG non-responders. Furthermore, as a group of functionally immature monocytes, Mono_MDSCs play a critical role in suppressing T cells by expressing high levels of inhibitory receptors (e.g., *PD-L1*) (37). Correspondingly, we observed an upregulation of *PD-L1* expression in IVIG non-responders (**Figure 6C**). In addition, we identified a monocyte subset (Mono_C1QA), which modulates the expression of complement components and receptors. Key complement activation pathway components, such as *C1QA*, *C1QB*, and *C1QC* (classical pathway) and *CFD* (alternative pathway), were upregulated in IVIG non-responders (**Figure 6D**). These results highlight the critical role of the complement system in IVIG non-responsive KD.

DEG analysis in monocytes demonstrated upregulation of canonical pro-inflammatory cytokines and chemokines (*CXCL10*, *CCL4*, *CXCL2*, *CCL4L2*, and *CCL3*), as well as CEBP family transcription factors (*CEBPB* and *CEBPD*), in IVIG non-responders (**Figure 6E**; **Supplementary Figure 10D**). *CEBPB* and *CEBPD* are key TFs regulating fatty acid metabolism-driven inflammation and are elevated in KD (38), suggesting that dysregulated fatty acid metabolism may contribute to IVIG non-responsiveness. GO enrichment analysis of upregulated DEGs showed that the terms “inflammatory response” and “cellular response to TNF” were enriched in IVIG non-responsive KD (**Figure 6F**), consistent with earlier inflammatory findings in this study (**Figure 2**). This further implies that monocytes may actively drive the heightened pro-inflammatory response in IVIG non-responders and thus mediate tissue damage. In line with the observations in other immune cells, the IFN-I-related pathways and genes (*ISG15*, *IFI27*, and *IFITM1/2/3*) in monocytes were upregulated in IVIG non-responders (**Figure 6G**, **Supplementary Figure 8**). GO terms associated with interferon signaling pathway (e.g., “type I interferon signaling pathway,” “response to interferon-α”) were also enriched in these patients (**Figure 6F**). Consistent with our scRNA-seq data, we found that plasma IFN-α levels showed an elevated trend in IVIG non-responders (**Supplementary Figure 10E**). To further validate our single-cell analysis results, we analyzed public bulk RNA-seq data and found that type I interferon-related pathways were upregulated in IVIG non-responsive patients (**Supplementary Figure 10F**). Among these, *IFITM1*, *IFITM3*, *IRF1*, and *IFNGR2* exhibited an increasing trend (**Supplementary Figure 10G**). Type I interferon augments TNF- and IL-1β-mediated inflammatory responses and is elevated in KD with coronary artery aneurysms (23, 39). Together, these findings support the hypothesis that type I interferon signaling may amplify S100A12–TLR4-driven inflammation, resulting in an exaggerated inflammatory response in IVIG non-responders.

Megakaryocytes contribute to inflammatory diseases and influence both platelet production and function (40). Platelet counts are widely used as a clinical indicator to predict IVIG treatment response (16, 41). Although megakaryocytes were enriched in IVIG non-responders, pathways related to platelet aggregation and activation (GO: 0070527 and GO: 0030168) were not upregulated, suggesting impaired platelet function in these patients (**Supplementary Figure 10H**).

## Discussion

In this study, we found that IVIG non-responsive KD was associated with significant alterations predominantly affecting pro-inflammatory cells and the adaptive immune system. Specifically, our study exhibited the enrichment of specific inflammatory cells (Mono_CD14, Mono_MDSCs, and megakaryocytes) along with naive and proliferative lymphocytes (CD8_Naive and NK_Pro), while effector lymphocyte subsets (CD4_Tfh, CD4_Treg, and CD8_Effector) showed a reduced trend. However, in the present study, the expansion of plasmablast and disrupted coordination between T- and B-cell responses may indicate humoral immune dysregulation in IVIG non-responsive KD, providing a novel research avenue. Moreover, the enhanced type I interferon response may account for the hyperinflammatory response and the high exhaustion of T and NK subsets in IVIG non-responders. To our knowledge, this is the first study to comprehensively report the mechanism of IVIG non-response using scRNA-seq. Our results revealed distinct cellular and transcriptomics features in IVIG non-responsive KD, providing underlying mechanisms and potential therapeutic strategies for this condition.

Elevated expression of multiple inflammatory cytokines is a hallmark of IVIG non-responsive KD patients (6–8). Our results also revealed a strong inflammatory cytokine response in IVIG non-responsive KD. Calgranulin family members, including *S100A8*, *S100A9*, and *S100A12*, were major contributors to this heightened inflammatory cytokine response. Although *S100A8/A9* was identified as the most highly expressed inflammatory mediator at the single-cell level, ELISA validation did not show significant differences. It is well-established that *S100A8/A9* exists as a dimer *in vivo* (42). Differences in molecular form, limited sample size, and methodological variations may have contributed to discrepancies in our ELISA results. Consistent with our findings, a previous clinical study has similarly reported increased *S100A12* and *MYD88* expression in IVIG non-responsive KD patients (8), further supporting the role of S100A12-mediated inflammatory signaling in IVIG resistance. Although likely influenced by the limited sample size, the ELISA results did not reach statistical significance. *S100A12* has been reported to activate pro-inflammatory responses via the TLR4–MyD88 signaling pathway, which in turn activates the NF-κβ pathway and induces the release of downstream inflammatory mediators including TNF-α and IL-1β (23). Enhanced TNF and TNF superfamilies (*TNFSF8* and *TNFSF10*) were also identified and validated in our study. TNF has been reported to be associated with IVIG non-response (43), and TNF-targeting drugs such as infliximab have been employed as therapeutic options for IVIG non-responders (44). Our previous work has identified *TNFSF10* as the major driver of the inflammatory storm in KD with CALs (13). TNFSF10 has been reported to mediate the overactivation of NK cells and cytotoxic T lymphocytes, a process that can be regulated by type I interferon (45, 46). Targeting TNFRSF10 may represent a promising therapeutic strategy for KD patients with IVIG non-response or CALs. We further identified the Mono_CD14_CD16 cell subset as the major source of inflammatory cytokines in IVIG non-responders. Collectively, targeting these cluster and cytokines may inhibit the downstream pro-inflammatory signal, providing novel therapeutic strategies for IVIG non-responders.

Our findings also highlight immune dysregulation and functional impairment of T/NK cells in IVIG non-responders. In our study, several naive-associated genes (*ICOS*, *NKG7*, *CCR7*, *TCF7*, *LEF1*, and *SELL*) exhibited increased expression trend in CD8^+^ T cells from IVIG non-responders. *ICOS*, a co-stimulatory immune checkpoint expressed on activated T cells, interacts with its ligand *ICOSL* to regulate multiple activities across distinct T-cell subpopulations, including activation and effector functions (47). These findings may suggest that CD8^+^ T cells in IVIG non-responders remain in a naive state and fail to perform their adaptive immune functions effectively. Furthermore, we observed that T and NK cells in IVIG non-responders exhibited a highly exhausted state, marked by increased inhibitory receptor expression and upregulation of exhaustion-related transcription factors, especially in the CD4_Treg, CD8_Effector, and NK_CD160 subsets. T-cell exhaustion is linked to ineffective adaptive immune response (23). Few prior studies have reported functional alterations in NK-cell subsets among IVIG non-responsive KD patients. Choi et al. reported that the proportion of CD56^−^CD16^+^ NK cells was significantly lower in IVIG non-responders than responders (48). However, this finding was not clearly observed in our study, likely due to the limited sample size. Nevertheless, our study revealed that NK cells in IVIG non-responders exhibited a highly exhausted phenotype, accompanied by elevated expression of MHC receptors, particularly within the NK_CD160 subset. Zhang et al. reported that the elevated expression of MHC-II in NK cells may reflect a highly activated state (49) and that excessive activation of MHC receptors also leads to NK-cell exhaustion (50). Highly exhausted NK cells may compromise their immune surveillance function, potentially contributing to a stronger immune-inflammatory response in IVIG non-responsive KD patients (51). Our previous research also demonstrated that IVIG combined with methylprednisolone could reduce NK-cell exhaustion (14). Thus, modulating overexpressed T/NK exhaustion could reverse dysfunction and restore effective immune responses. Furthermore, high expression of cytotoxic molecules (e.g., *PRF1*, *GZMA*, and *GNLY*) in T/NK cells may induce immunopathology by triggering inflammatory responses (23), further contributing to the immunopathology of IVIG non-responders. Herein, the dysregulated T-/NK-cell immune response may be a contributing factor to IVIG non-responsive KD.

IFN-β has been proposed as an effective biomarker for KD identification (52). Interferon genetic polymorphism and serum IFN-γ level have also been reported to be associated with KD susceptibility and IVIG responsiveness (53, 54). In our study, interferon-related gene alterations in IVIG non-responders were consistently observed across scRNA-seq, bulk RNA-seq, and ELISA analyses. Wilson et al. and Crow et al. reported that sustained type I interferon responses may contribute to the pathogenesis of chronic viral infections and autoimmune diseases (55, 56). The potential mechanisms may include a sustained type I interferon response leading to CD8^+^ T-cell exhaustion (28), enhanced IFN-γ production by NK cells (29), and an excessive inflammation mediated by TNF-α and IL-1β (23, 39). Although, likely due to the limited sample size, the differences were not statistically significant between the IVIG responsive and non-responsive groups, these findings provide valuable insights and directions for future research. Upregulation of interferon-related genes in the lungs and coronary arteries of KD and the presence of virus-like particles near KD inclusions in ciliated bronchial epithelium support the hypothesis that KD may result from a ubiquitous viral factor (39). Building upon these findings, it can be inferred that IFN-I and related components of the IFN pathway might be potential therapeutic targets.

In this study, we also found that Mono_C1QA exhibited elevated expression of upstream components in both the classical and alternative complement activation pathways in IVIG non-responders. This suggests that the complement system can be activated in IVIG non-responsive KD, although the underlying mechanisms warrant further investigation. Mono-MDSCs, functioning as a heterogeneous population of immature monocytic cells, were significantly enriched in IVIG non-responsive KD. These cells exert potent immunosuppressive effects by expressing elevated levels of inhibitory receptors (PD-L1) (37), which play a pivotal role in suppressing T-cell function. Thus, we hypothesize that it may contribute to immune suppression in IVIG non-responders. Beyond the findings in Mono-MDSCs, we also identified impaired antigen-presenting and phagocytosis capacity in mDCs, further indicating peripheral immune paralysis in IVIG non-responsive KD.

There are several limitations in the present study. First, our analysis relied on frozen blood samples. Since it is challenging to determine IVIG responsiveness before treatment, we pre-froze PBMC samples and then thawed eligible samples according to IVIG responsiveness, which might lead to the loss of certain immune cells, potentially affecting our results. Second, the sample size of this study was relatively small, derived from a single ethnic population and lacked validation in an independent cohort. Third, our findings suggested that the complement system and type I interferon signaling may offer important insights into the mechanisms underlying IVIG non-response; however, further verification is required. These limitations may restrict the generalizability of our conclusions.

In conclusion, our study presents a peripheral immune response profile for IVIG treatment in KD, highlighting changes in immune cell proportions and functions. IVIG non-responsive KD exhibited a stronger inflammatory cytokine response, mainly driven by the Mono_CD14_CD16 subset and T/NK-cell dysfunction. We propose that type I interferon signaling is a key upstream regulator of these changes. Upon validation, the expression levels of five cytokines (*S100A8/A9*, *S100A12*, *TNF*, *TNFSF8*, and *TNFSF10*) and interferon-α were consistent with the single-cell analysis results. Taken together, these findings will advance our understanding of the mechanisms underlying IVIG non-responsiveness and provide valuable insights for developing effective clinical treatments.

## Data Availability

All data are publicly available at the China National Center for Bioinformation with accession number OMIX012638.
