# Supplementary material for: Single-cell transcriptomic analysis reveals a systemic immune dysregulation in intravenous immunoglobulin non-responsive Kawasaki disease
Source: Front Immunol. 2025 Nov 27;16:1702290. doi: 10.3389/fimmu.2025.1702290 (PMC12695851; doi:10.3389/fimmu.2025.1702290)
Supplement: Supplementary file 1 [file DataSheet1.pdf]

Supplemental Materials for

Single-cell transcriptomic analysis reveals a systemic immune dysregulation in intravenous immunoglobulin non-responsive Kawasaki disease

Chenhui Feng[1,2,†], Qirui Li[3,†], Minna Yang[1,2], Yeshi Chen[1,4], Mingming Zhang[1], Hongmao Wang[1], Xiaohui Li[1,5*]

*** Correspondence to:**

Xiaohui Li, MD, PhD.

Department of Cardiovascular Medicine, Capital Center for Children's Health, Capital Medical University, No.2, Yabao Road, Chaoyang District, Beijing, China

Email: lxhmaggie@pumc.edu.cn

[+] These authors have contributed equally to this work.

The file includes: Supplementary Figure 1-10 with their legends, Supplementary Table 1-3

Supplementary Figure 1-10 with their legends

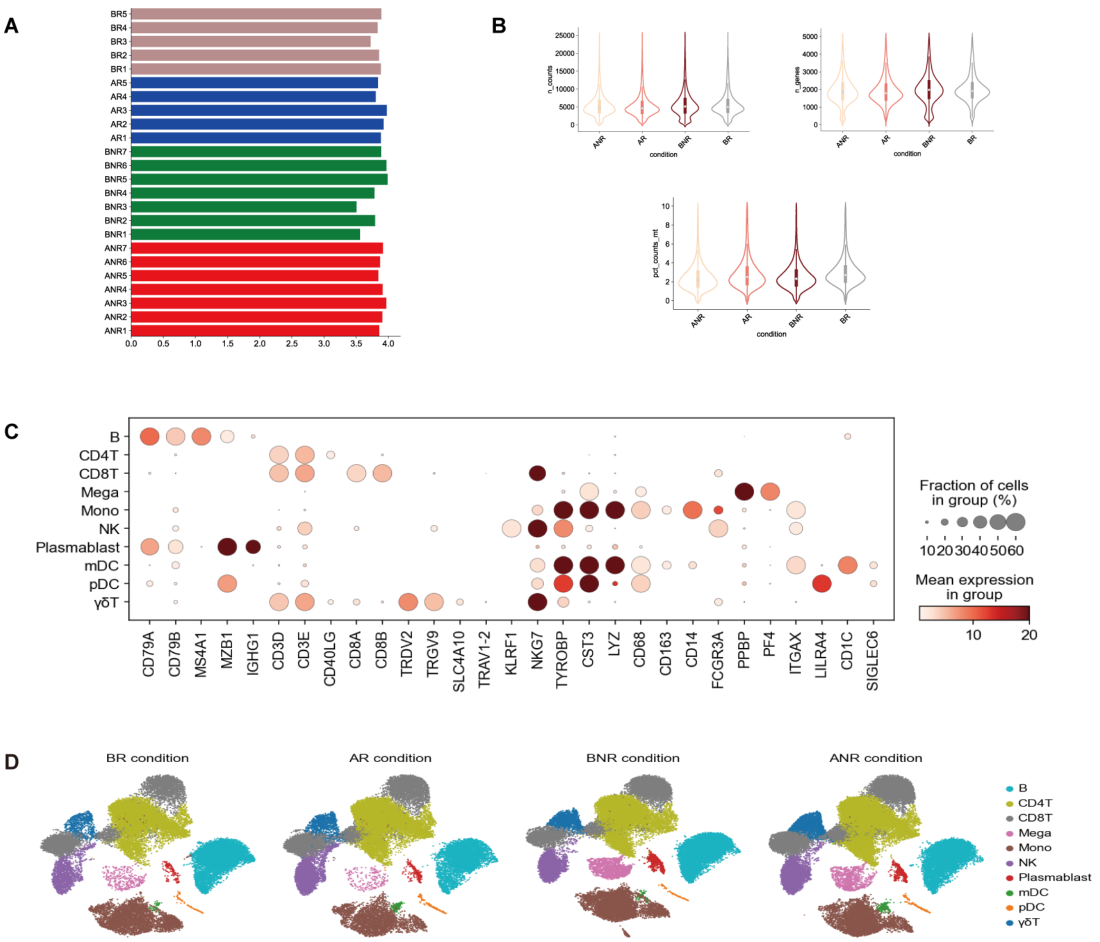

Supplementary Figure 1. Characteristics of the single-cell transcriptional profiles of PBMCs from 24 subjects, related to Figure 1. (A) Box plots illustrating the log10 transformed number of cells for each sample. 5 BR samples were obtained from IVIG responsive KD patients before treatment, 5 AR samples were obtained from IVIG responsive KD patients after treatment, 7 BNR samples were obtained from IVIG non-responsive KD patients before treatment, 7 ANR samples were obtained from IVIG non-responsive KD patients after treatment. **(B)** Distribution of the unique molecular identifier (UMI) counts per cell, gene counts per cell, and percentage of mitochondrial transcripts per cell detected for cells in each group. The error bars represent Standard Error (SE). **(C)** Dot plot showing the expression of canonical cell

markers for each cell type. **(D)** The UMAP projection for the four conditions on different panels. Cells are colored by the 10 major cell types.

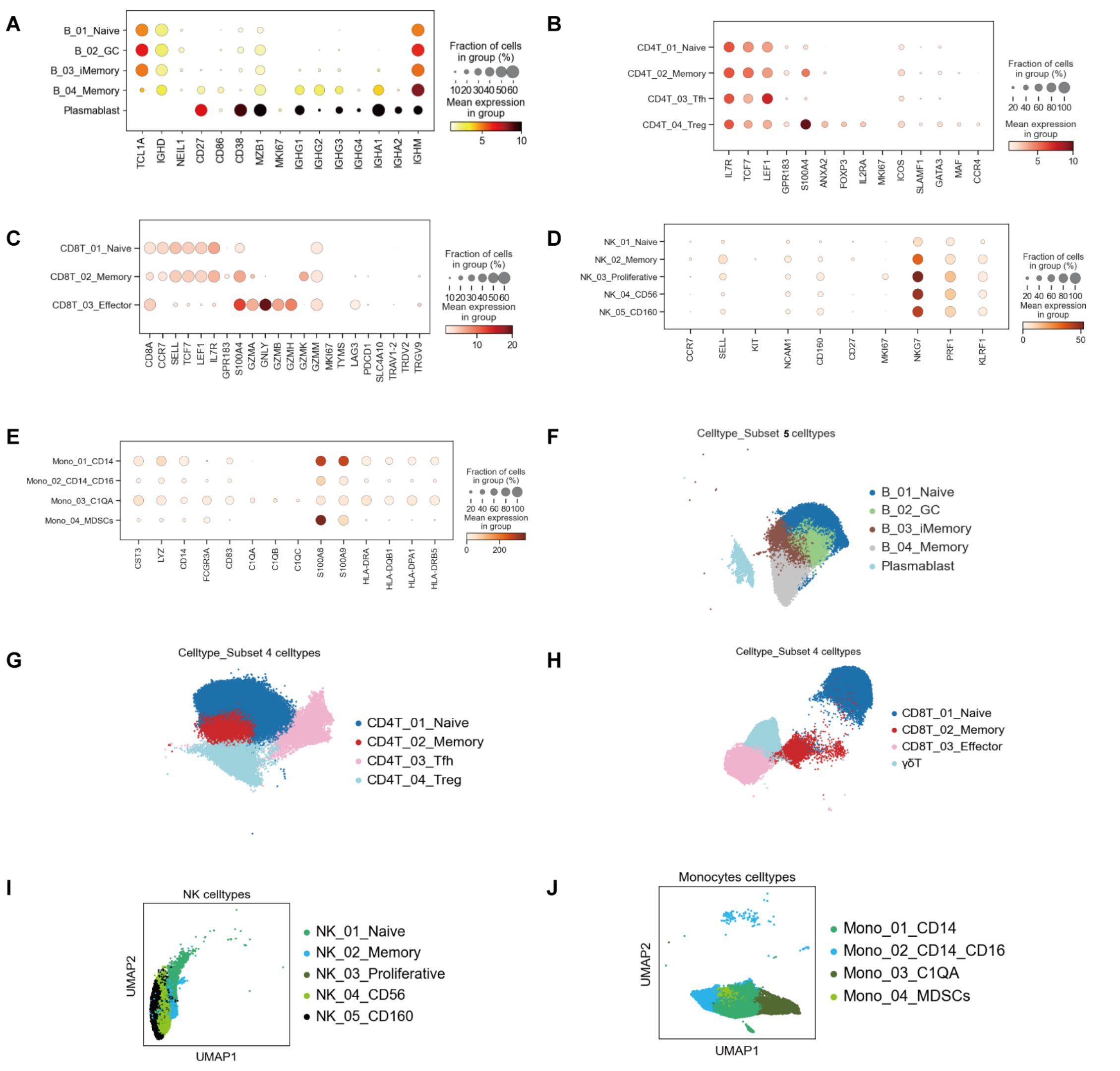

Supplementary Figure 2. Characteristics of the single-cell transcriptional profiles of PBMCs from 24 subjects, related to Figure 1. (A-E) Dot plots of selected marker genes (Rows) for cell subsets (Columns) within each cell lineage, including B, CD4^{+}T, CD8^{+}T, NK and monocyte cell subsets. **(F-J)** The clustering result of B, CD4^{+}T , CD8^{+}T , NK and monocyte cell subsets. Each point represents one single cell, colored according to cell type.

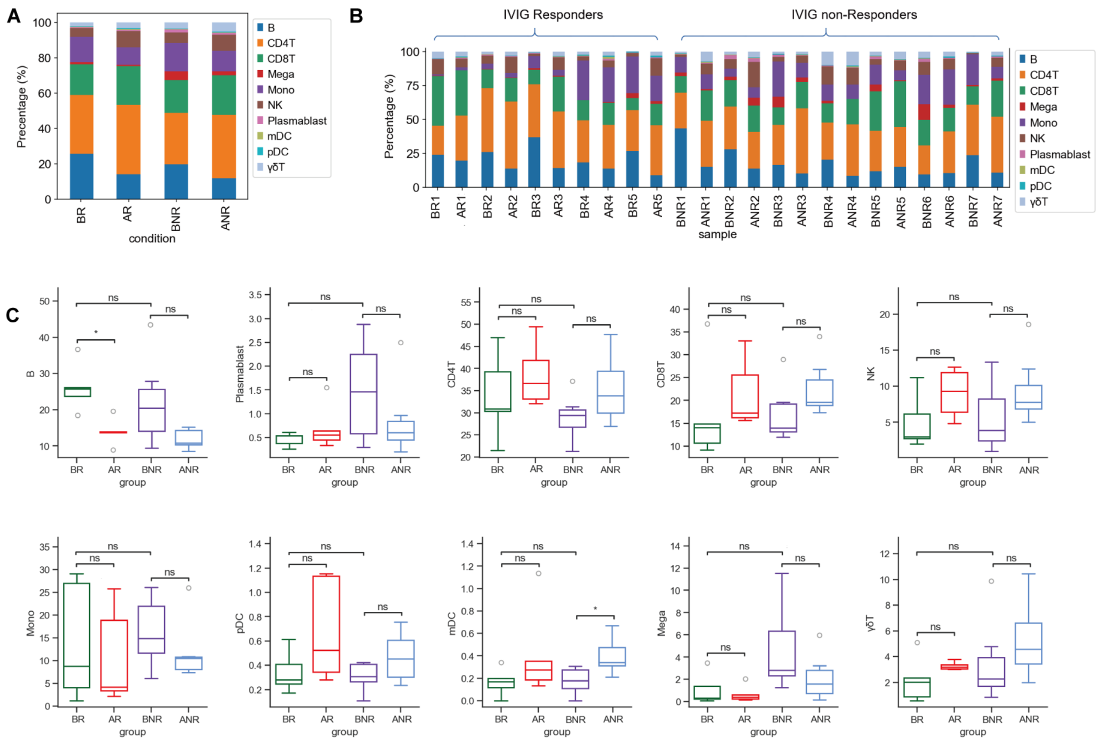

Supplementary Figure 3. Detailed data output and visualization of single-cell transcriptomic profiling of PBMCs from 24 samples, related to Figure 1. (A) Stack bar plot showing the relative proportion of 10 cell subtype for each of the 24 samples. **(B)** Stacked bar plot showing the relative proportion of 10 cell subtype derived from BR, AR, BNR and ANR groups. **(C)** The distribution of each immune cell type across 4 conditions. The y-axis shows the average percentage of each immune cell type. Conditions are displayed in different colors on the x axis.

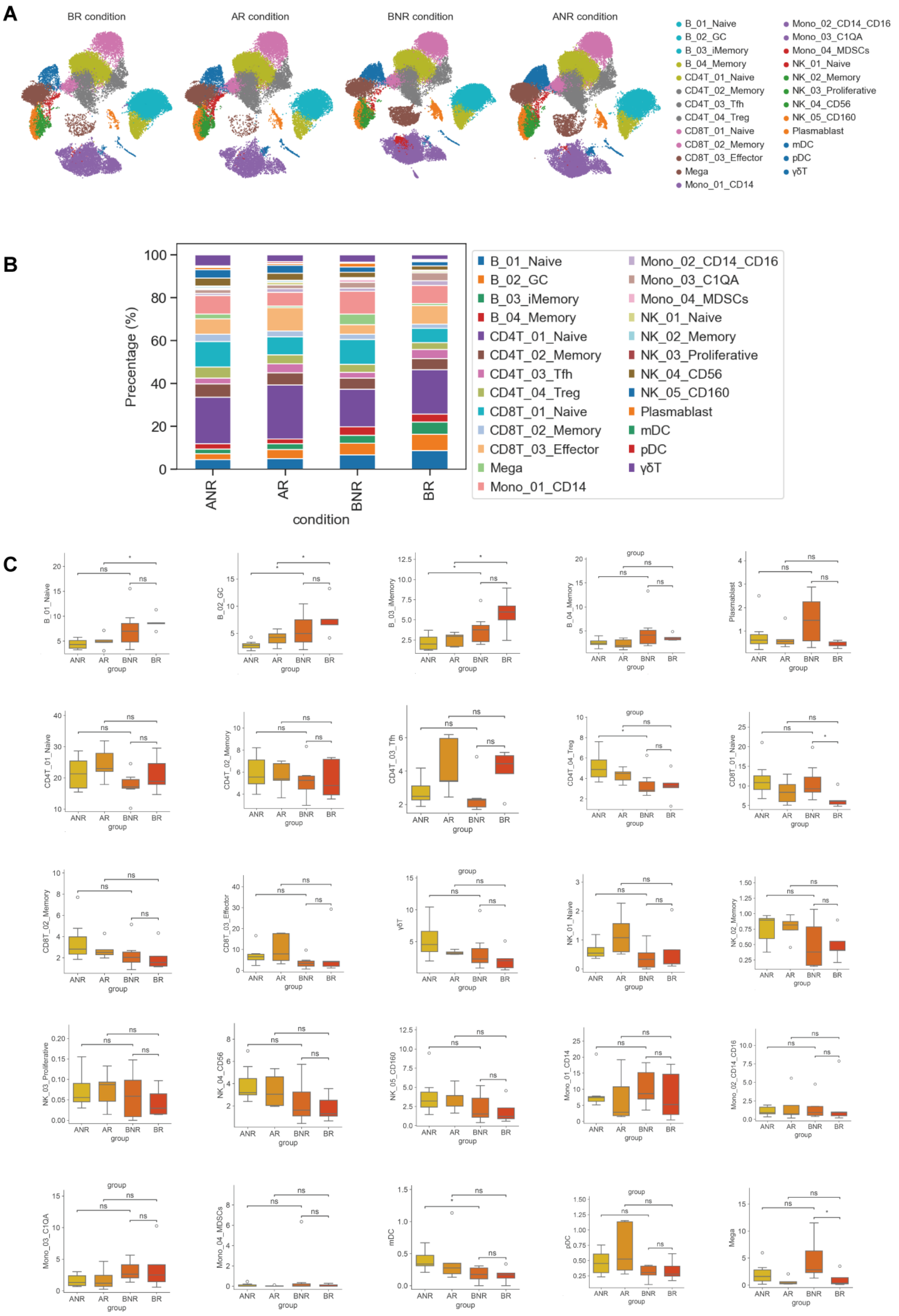

Supplementary Figure 4. Detailed data output and visualization of single-cell transcriptomic profiling of PBMCs from 24 samples, related to Figure 1. (A) Identification of peripheral blood mononuclear cells (PBMC) subcluster in different groups. **(B)** Comparing the proportion of different cell subcluster across different groups. **(C)** Box plots comparing the proportion of different cell subcluster across different groups.

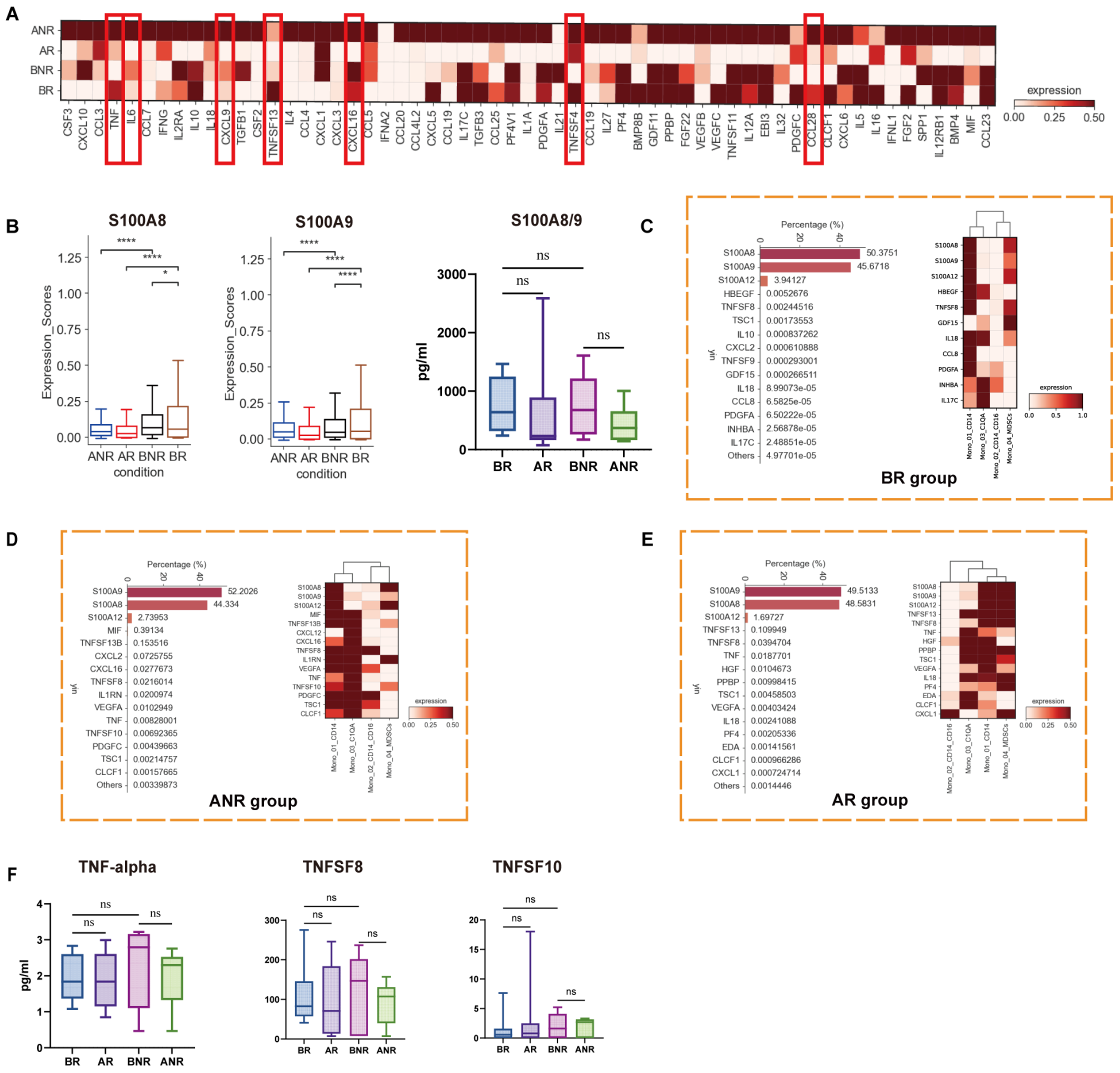

Supplementary Figure 5. Details of inflammatory cytokine response associated in different groups. (A) Heatmap portrayed the expression of individual cytokines in monocyte from 4 conditions. **(B)** Box plots showing the levels of S100A8/9 in monocyte by sc-RNA seq and ELISA. **(C)** Bar chart showing the relative contribution of the top 15 cytokines in IVIG responders before treatment and the heatmap illustrating the expression of these 15 cytokines within each monocyte subtype. **(D)** Bar chart depicting the relative contribution of the top 15 cytokines in IVIG non-responders after treatment and the heatmap showing the expression of these cytokines within each monocyte subtype. **(E)** Bar chart depicting the relative

contribution of the top 15 cytokines in IVIG responders after treatment and the heatmap showing the expression of these cytokines within each monocyte subtype. **(F)** Box plots showing the levels of TNF-α, TNFSF8 and TNFSF10 in each group by ELISA. Significant differences were determined by Two-sided Student's *T*-test with Bonferroni correction (*p<0.05, **p<0.01, ***p<0.001, ****p<0.0001, ns p>0.05).

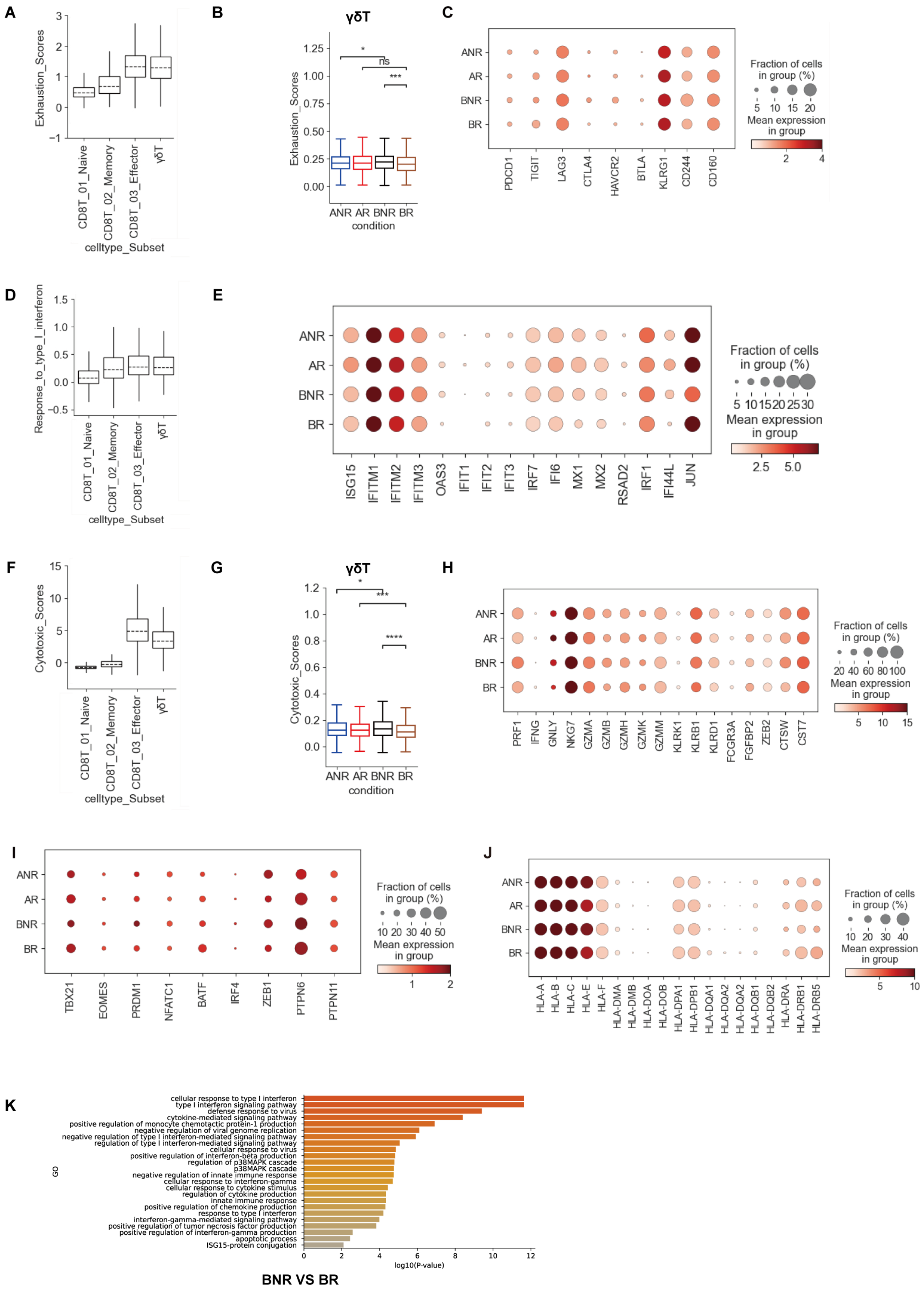

Supplementary Figure 6. Characterization of gene expression differences in T cells across different groups. (A) Box plot showing the contribution of different CD8^{+} T cell types to the exhaustion score. **(B)** Box plots showing the exhaustion

scores in γδT cell between different groups. **(C)** Dot plot demonstrating the expression of selected exhaustion genes in γδT cells between different conditions. **(D)** Box plot showing the contribution of different $CD8^+$ T cell types to the IFN-I signaling pathway score. **(E)** Dot plot showing the expression of IFN-I-related genes in γδT cells between different conditions. **(F)** Box plot showing the contribution of different T cell types and γδT cells to the cytotoxicity score. **(G)** Box plots showing the cytotoxicity scores in γδT cell between different groups. **(H)** Dot plot demonstrating the expression of selected cytotoxicity genes in γδT cells between different conditions. **(I)** The expression of genes associated with proliferation, differentiation and function in $CD8^+$ T cell across different groups. **(J)** Dot plot showing the expression of genes associated with HLA-I and HLA-II genes in $CD8^+$ T cell. **(K)** GO enrichment analysis of DEGs in IVIG non-responder compared with IVIG responder before treatment in $CD8^+$ T cell. DEGs refer to genes with Wilcoxon adjusted *p* value <0.05.

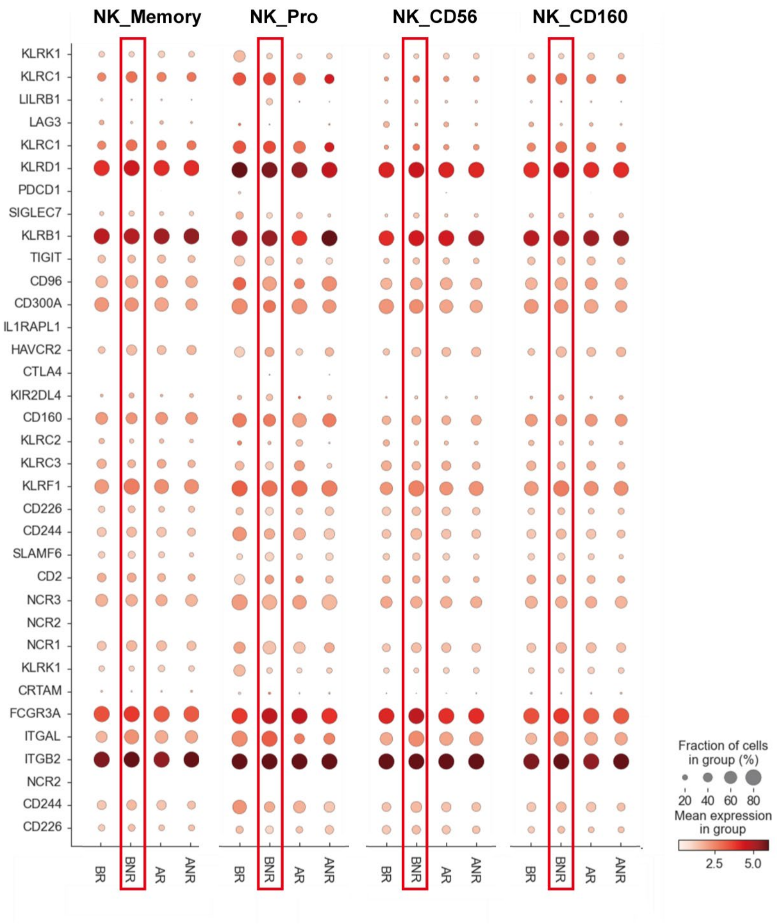

Supplementary Figure 7. Immunological features of NK cell subsets. Dot plots showing the expression of activation-related genes in each NK cell subtype per condition.

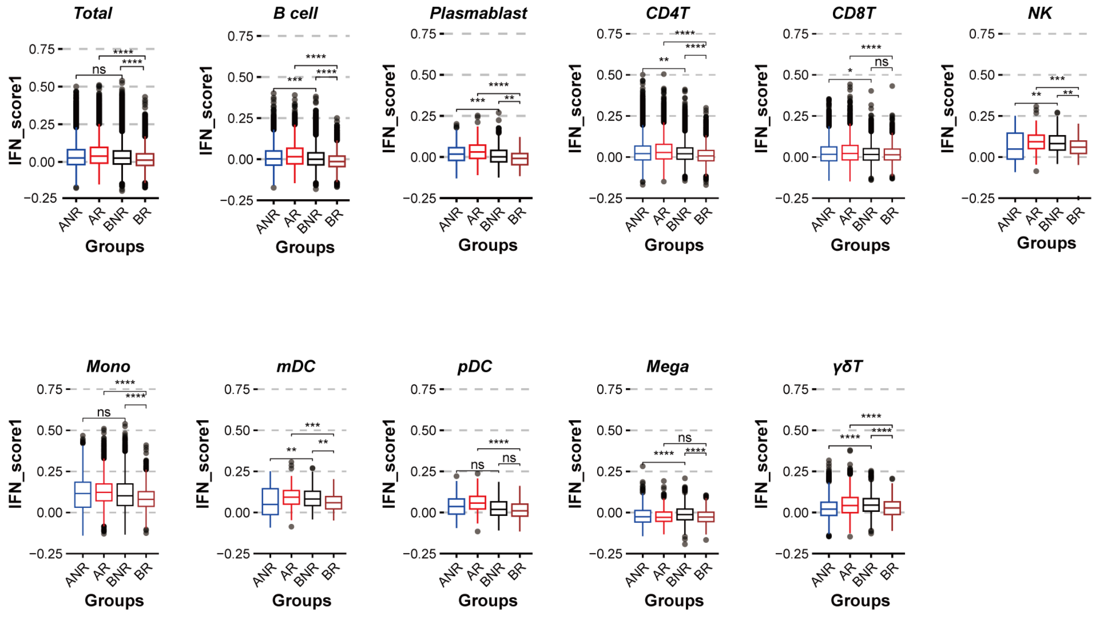

Supplementary Figure 8. IFN-I features of total and each cell cluster. Box plots of IFN-I score of total and each cell cluster between different groups.

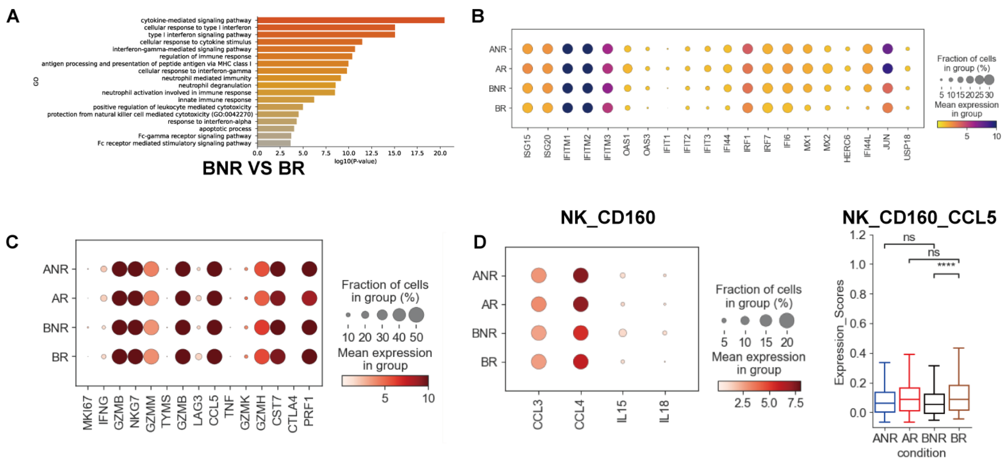

Supplementary Figure 9. Immunological features of NK cell subsets. (A) GO enrichment analysis of DEGs in IVIG non-responder compared with IVIG responder before treatment in NK cells. DEGs refer to genes with Wilcoxon adjusted *p* value <0.05. **(B)** Dot plot showing the expression of genes associated with type I IFN-related genes in NK cell. **(C)** The dot plot showing the expression of selected DEG genes in NK cell between different groups. **(D)** Dot plot and box plot depicting expression for NK-produced cytokines in NK_CD160 cell subtype between different groups.

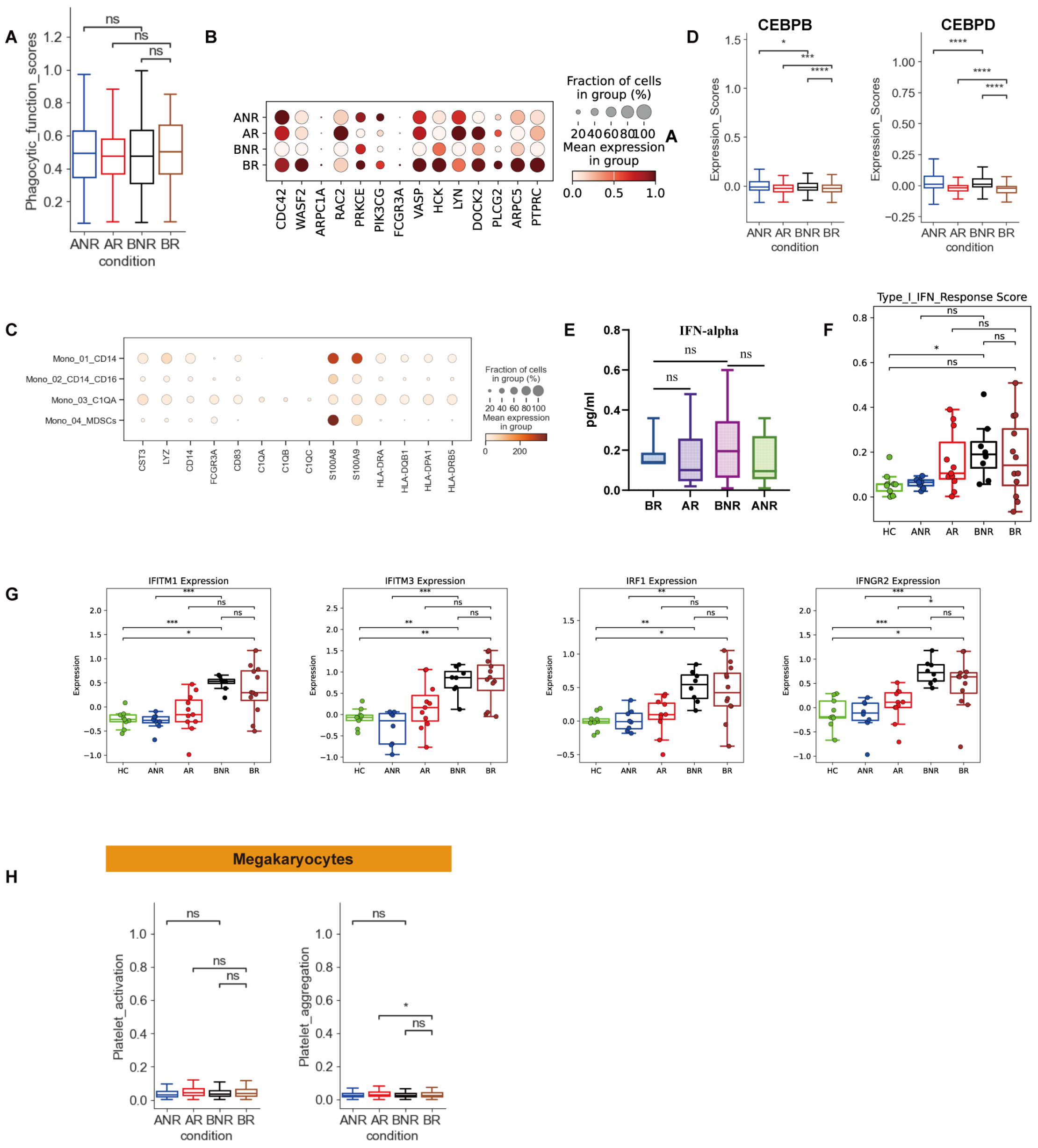

Supplementary Figure 10. Dysregulation of myeloid cell functions in IVIG non-responsive KD. (A) Box plots of phagocytosis function score of mDCs between different groups. **(B)** Dot plots depicting the expression of phagocytosis-related genes in mDCs. **(C)** Dot plots of selected marker genes (Rows) for cell subsets (Columns) within monocyte. **(D)** Box plots showing the expression of CEBPB and CEBPD in monocyte. **(E)** Box plots showing the expression levels of IFN-alpha by ELISA. **(F)** Box plots of Type_I_IFN_Response score in bulk RNA-seq level between different

groups (HC, BR, AR, BNR and ANR). Significant differences were determined by Two-sided Student's *T*-test with Bonferroni correction (*$p<0.05$, **$p<0.01$, ***$p<0.001$, ****$p<0.0001$, ns $p>0.05$). **(G)** Box plots showing the expression level of IFITM1, IFITM3, IRF1 and IFNGR2 in bulk RNA-seq dataset between different groups (HC, BR, AR, BNR and ANR). **(H)** Box plots showing the expression of platelet aggregation (left) and platelet activation (right) in megakaryocyte.

HC, healthy control; KD, Kawasaki disease; BR: IVIG responders before IVIG treatment; AR: IVIG responders after IVIG treatment. BNR: IVIG non-responders before IVIG treatment; ANR: IVIG non-responders after IVIG treatment.

Table S1 (Sheet 1). Marker genes and signature genes for 10 major celltypes, related to Fig 1, Fig 2, Fig S1, Fig S2 and Fig S3.		
**Name of Major celltypes**	**Canonical genes**	**Signature genes**
**B cells**	CD79A, CD79B, MS4A1	CD79A HLA-DRA MS4A1 HLA-DQA1 HLA-DRB1 HLA-DQB1 CD79B BANK1 LINC00926 RALGPS2 CD74 HLA-DMA IGHM MEF2C NIBAN3 AFF3 CD83 NCF1 HVCN1 LTB CD37 SWAP70 HLA-DMB TNFRSF13C SNX2 IRF8 FCER2 FCRL1 SYNGR2 TCL1A CXXC5 FCRLA IGKC EAF2 CD22 POU2AF1 FCMR CD19 P2RX5 BLK LYN SPIB CYB561A3 VPREB3 BLNK TCF4 PLPP5 HLA-DRB5 GNG7 BACH2
**Plasmablast**	MKI67	TXNDC5 MZB1 JCHAIN SEC11C TNFRSF17 FKBP11 PDIA4 UBE2J1 AQP3 PRDM1 SSR3 SDF2L1 DERL3 MYDGF ITM2C TXNDC11 CD27 SELENOS IGHA1 MANF LMAN1 SEC61A1 CD59 MAN1A1 CLPTM1L OSTC ZBP1 FKBP2 PRDX4 KDELR2 CD38 SLAMF7 ELL2 B4GALT3 ERLEC1 CRELD2 WARS ERN1 CYTOR TRIB1 DNAJB11 RRBP1 GMPPB CCDC167 FNDC3B SEMA4A SRPRB HDLBP CHPF ARF4 XBP1
**CD4+T cells**	CD3D, CD3E, CD40LG	LTB IL7R TCF7 LEF1 MAL NOSIP RCAN3 EEF1A1 PIK3IP1 CAMK4 AQP3 FLT3LG TMEM123 TPT1 CCR7 NDFIP1 PRKCQ-AS1 SELL FOXP1 PIM2 GIMAP5 GPR183 BCL11B CD27 CD28 TRAT1 PBXIP1 DGKA SOCS3 MCUB SNHG8 INPP4B CD5 MYC S1PR1 CD6 ABLIM1 IL6ST MGAT4A CDKN1B GIMAP7 OXNAD1 BCL2 TRABD2A TESPA1 TTC39C FCMR LINC02273 ARHGAP15 CD4

**CD8+T cells**	CD3D, CD3E, CD8A, CD8B	CD8A CD8B GZMH CD3G GZMA KLRK1 CTSW FGFBP2 PRF1 C12orf75 LINC02446 CD2 FCRL6 DUSP2 LAT SYNE1 THEMIS PPP2R5C SAMD3 PAXX GZMB MATK KLRG1 ADGRG1 ZAP70 CD6 CCL4 SYNE2 KLRD1 SSBP4 LYAR PRKCH TBX21 YWHAQ CCNH TGFBR3 TPST2 PYHIN1 GNG2 ITGB7 CLEC2D APMAP ZEB2 S1PR5 ZBTB38 SKAP1 ARPC5L LAG3 CTSC CLEC2B
**γδT**	TRDV2, TRGV9	TRDV2 TRGV9 KLRG1 KLRB1 DUSP2 CTSW KLRD1 GZMA TRBC1 CCL4 PRF1 CD247 GZMH MATK TRDC KLRK1 CD3G SPOCK2 HOPX GZMB KLRC1 LAG3 RORA SYNE2 LYAR PPP2R5C GNG2 ALOX5AP PMAIP1 PTMS FGFBP2 TGFBR3 SAMD3 SYNE1 PAXX ZBTB16 BHLHE40 TBX21 S1PR5 SH2D2A ADGRG1 TRGC1 ERN1 NCR3 APMAP IER5 TRAC LBH PIK3R1
**NK**	KLRF1, NKG7,TYROBP	PRF1 GNLY GZMB CD247 CTSW FCGR3A SPON2 KLRD1 GZMA TYROBP FGFBP2 KLRF1 HOPX CD7 S1PR5 CLIC3 GZMH IL2RB CCL4 ADGRG1 TRBC1 KLRB1 MATK GNG2 RHOC KLRK1 SH2D1B PRSS23 CD63 CTSD APMAP TBX21 TTC38 FCER1G ZAP70 ZEB2 PLAC8 PLEK SPN IFITM2 CX3CR1 TRDC KLRC2 BHLHE40 CD300A FCRL6 DHRS7 NCR3 METRNL AOAH
**Monocytes**	CST3, LYZ, CD14 and/or CD16	IFI30 CTSS AIF1 FCN1 CST3 TYROBP S100A11 LYZ S100A9 SPI1 LST1 SERPINA1 FCER1G S100A8 CD68 TKT BRI3 TSPO CFD CYBB MNDA MAFB NPC2 TYMP LGALS3 GRN NEAT1 NCF2 CSTA HLA-DRA CEBPD PYCARD FGL2 CD14 RNF130 IFITM3 CFP CTSZ FPR1 PILRA PLAUR CLEC7A ANXA5 TALDO1 IGSF6 HCK MS4A6A VCAN HLA-DRB1 NCF1

**pDC**	LILRA4, ITM2C	ITM2C TCF4 PPP1R14B PLD4 MZB1 IRF8 CCDC50 LILRA4 STMN1 JCHAIN C12orf75 HLA-DRA SERPINF1 APP VEGFB GZMB IRF7 UGCG BCL11A CLIC3 IL3RA CYB561A3 TSPAN13 HERPUD1 HLA-DMA NIBAN3 HLA-DPB1 CLEC4C IGKC HLA-DPA1 PEBP1 GAS6 LRRC26 RUNX2 BLNK SPIB CXCR3 XBP1 DERL3 OSTC OFD1 TPM2 SELENOS TRAF4 RNASE6 SCT PAXX PPM1K FCHSD2 TP53I13
**mDC**	CD1c	HLA-DRA HLA-DPB1 HLA-DRB5 HLA-DRB1 HLA-DQB1 HLA-DPA1 HLA-DQA1 HLA-DMA CST3 FCER1A PLD4 HLA-DMB ITGB7 PEBP1 LGALS2 CPVL CD1C CIITA RTN1 CLEC10A PPA1 CTSH APEX1 CCDC88A TMEM14C HMGA1 HLA-DQA2 RNH1 ENHO NDRG2 MAP4K1 SPINT2 UVRAG DDOST ANXA2 GSTP1 LY86 TMEM109 C1QBP FLT3 KLF10 SYNGR2 KCNK6 PEA15 CTSZ PRCP OSTC HLA-DOA HDAC9 MEF2C
**Megakaryocyte**	CST3, PPBP, PF4	TUBB1 NRGN CAVIN2 PPBP PF4 GNG11 GP9 PRKAR2B TUBA4A SPARC RGS18 RGS10 HIST1H2AC MPIG6B LIMS1 TPM4 CTSA MPP1 F13A1 CMTM5 GRAP2 TRIM58 GP1BB ODC1 MAX YWHAH MTURN KIF2A TMEM40 CALM3 CLU NAP1L1 TLN1 FERMT3 PTGS1 ITGA2B VCL TSC22D1 ILK ITGB3 MARCH2 ESAM ACTN1 GSTO1 C2orf88 TSPAN33 PTPN18 GPX4 TREML1 PDLIM1

**Table S1 (Sheet 2). Marker genes and signature genes for B cell subtypes, related to Fig 1, Fig 2, Fig S1, Fig S2 and Fig S3.**		
**Name of B cell subtypes**	**Canonical genes**	**Signature genes**
**B_Naive**	TCL1A, IGHD	TCL1A FCER2 IGHM IGHD BACH2 PLPP5 IL4R CD79A YBX3 FCRL1 CD37 CD79B HVCN1 AFF3 CD74 LINC00926 NIBAN3 NCF1 CD83 MEF2C CD22 CAMK2D HLA-DRB1 COL19A1 APLP2 CD72 HLA-DRA CALHM6 MS4A1 FOXO1 PLEKHA2 HLA-DQB1 NCK2 ZCCHC7 HLA-DQA1 VPREB3 TAPT1 HLA-DOB ZNF318 SNX2 TSPAN13 BIRC3 CCR7 ADK RUBCNL PAX5 HLA-DMA SNX29 BCL7A EAF2
**B_GC**	IGHD, NEIL1	PLD4 ITM2C GPX1 NRGN TCL1A VPREB3 PPBP NIBAN3 LILRA4 FCRLA SPIB MZB1 CD72 JUP NPC2 MYO7B CYB561A3 GNG11 CD79A PPP1R14A ALOX5 SPARC CD79B NEIL1 ATP2A3 CAVIN2 PRKAR2B LIMS2 IGHD CCL5 IFITM3 CLU TUBB1 PDLIM1 DTX1 IMPDH2 MYL9 EEF1A1 PF4 RGS19 CCDC50 PPP1R14B SOX4 AEBP1 ARMH1 H2AC6 LYL1 PTEN MPIG6B NCF1
**B_iMemroy**	IGHD, CD27	NRGN PPBP TUBB1 GNG11 CLU CAVIN2 SPARC PRKAR2B F13A1 PF4 MYL9 MARCKS MPIG6B GP1BB AIM2 SCIMP IGHG2 ITGA2B CTSA TREML1 MAP3K7CL GRAP2 CD82 GP9 TIMP1 IER5 RGS18 MTURN LINC01857 ITGB3 VCL LIMS1 CTSH IFI30 GNLY CD27 YWHAH FOSB PTGS1 GPR183 ODC1 CDKN1A TPM4 BCL2A1 CMTM5 PGRMC1 SELENOM NLRC5 ARHGAP24 CDC42EP3
**B_Memory**	CD27	TNFRSF13B IGHA1 MARCKS IGHG2 CD82 LINC01781 POU2AF1 CD27 CPNE5 BLK VOPP1 AIM2 HIPK2 ACP5 IGHG1 SCIMP SMARCB1 IFI30 CLECL1 COCH IER5 BANK1 UBE2N BASP1 JCHAIN CAPG UBE2J1 ARHGAP25 GPR183 SPIB CCDC50 NEK6 CTSH IGHA2 ZFAND6 TOR3A HSH2D MKNK2

		PTPN1 TMEM154 CRIP2 PVT1 ATP2B1 CD24 SP140 ANKRD13A ANXA4 RILPL2 NLRC5 ZBTB38
**B_Plasmablast**	MKI67	TXNDC5 MZB1 JCHAIN SEC11C TNFRSF17 FKBP11 PDIA4 UBE2J1 AQP3 PRDM1 SSR3 SDF2L1 DERL3 MYDGF ITM2C TXNDC11 CD27 SELENOS IGHA1 MANF LMAN1 SEC61A1 CD59 MAN1A1 CLPTM1L OSTC ZBP1 FKBP2 PRDX4 KDELR2 CD38 SLAMF7 ELL2 B4GALT3 ERLEC1 CRELD2 WARS ERN1 CYTOR TRIB1 DNAJB11 RRBP1 GMPPB CCDC167 FNDC3B SEMA4A SRPRB HDLBP CHPF ARF4

Table S1 (Sheet 3). Marker genes and signature genes for CD4+T cell subtypes, related to Fig 1, Fig 2, Fig S1, Fig S2 and Fig S3.		
**Name of CD4+T cell subtypes**	**Canonical genes**	**Signature genes**
**CD4_Naive**	CCR7, SELL	LEF1 CCR7 MAL EEF1A1 PRKCQ-AS1 IL6ST EEF1B2 ACTN1 TRABD2A TPT1 TSHZ2 ABLIM1 AIF1 PCED1B CAMK4 OXNAD1 MYC RCAN3 BACH2 C1orf162 MAML2 SARAF SATB1 PDE3B CHRM3-AS2 SOCS3 LDLRAP1 SNHG7 NELL2 NUCB2 CD27 TXK TMIGD2 AK5 EPHX2 EEF1G CITED4 ADTRP BEX3 GIMAP1 HIST1H1D RETREG1 SH3YL1 LINC01550 LINC00402 APBA2 SESN3 AC243960.1 ARMH1 TCEA3
**CD4_Memory**	GPR183, S100A4	NRGN PPBP SPARC CAVIN2 TUBB1 CLU PRKAR2B MYL9 GNG11 PF4 F13A1 GP1BB MPIG6B HIST1H2AC ITGA2B TREML1 CTSA NCOA4 MTURN GP9 PGRMC1 MAP3K7CL RGS18 VCL PTGS1 ACTN1 CST3 ITGB3 AP001189.1 PF4V1 MARCH2 GRAP2 TPM4 BEX3 MMD TIMP1 CMTM5 RNF11 SNN AP003068.2 HIST1H3H LIMS1 LEF1 MPP1 THBS1 TSC22D1 AP000547.3 SNCA PDLIM1 CCR7

**CD4_Tfh**	ICOS, SLAMF1	STMN1 IL7R SOX4 CAMK4 SATB1 LRRN3 LEF1 CCR7 BACH2 ABLIM1 TCF7 ACTN1 ITGA6 PDE7A TRABD2A BEX3 LINC00861 NUCB2 MARCKSL1 SERPINE2 CHI3L2 FCGRT IL6ST TGFBR2 SSBP2 NACA RBMS1 EPHX2 SATB1-AS1 BCL11B RETREG1 MAML2 H2AC6 ITM2A SARAF RGS10 AIF1 EEF1A1 PIK3IP1 MAL GRAP2 BBC3 CRLF3 LEF1-AS1 PECAM1 TMIGD2 EPHB6 NAA16 CHMP7 FOXP1
**CD4_Treg**	FOXP3, IL2RA	CTLA4 FOXP3 TIGIT RGS1 DUSP4 CYTOR IL32 IKZF2 ARID5B CD27 STAM CLDND1 SHMT2 RTKN2 MIR4435-2HG IL2RA PMAIP1 SLFN5 TTN TENT5C HLA-DQB1 PRDM1 LGALS3 CCM2 CHST11 NIBAN1 FAS TPM4 PYHIN1 PELI1 OGDH JPT1 EPSTI1 SIT1 LIMS1 TOX HMGB2 ELOVL5 CMTM7 IL2RB GLRX CD28 MXD4 HLA-DMA TRAC IFI16 CORO1B CASK CDC25B LGALS9

**Table S1 (Sheet 4). Marker genes and signature genes for CD8+T cell subtypes, related to Fig 1, Fig 2, Fig S1, Fig S2 and Fig S3.**		
**Name of CD8+T cell subtypes**	**Canonical genes**	**Signature genes**
**CD8_Naive**	CCR7, SELL	LEF1 CCR7 NELL2 EEF1A1 RCAN3 MAL LINC02446 PRKCQ-AS1 ACTN1 CD8B AIF1 CAMK4 OXNAD1 TRABD2A EEF1B2 PDE3B CD27 ABLIM1 LDLRAP1 NUCB2 BACH2 MYC SATB1 PCED1B MAML2 SH3YL1 ARMH1 TPT1 DGKA BCL2 SPINT2 NPM1 EEF1G SARAF SERINC5 TESPA1 TXK IL6ST SNHG7 NT5E RETREG1 LRRN3 LSR AQP3 IMPDH2 TMIGD2 RACK1 A1BG CITED4 CARS
**CD8_Memory**	GPR183, S100A4	GZMK FOSB RGCC NELL2 GPR183 RCAN3 CD27 CAMK4 CSRNP1 HOOK2 LEF1 SOCS3 CD28 TSPYL2 CLDND1 YBX3 EEF1A1 AQP3 ARL4A TSPYL1 ZNF331 RGS1 LMNA NCK2 CCR7 PAG1 TTC39C DGKA GATA3 LSR CD82 FAM102A FXYD2 PDE4B TRADD TNFSF8 INPP4B CD8B LDLRAP1 BCL2 MAL CASK LINC02273 AC020916.1 LYAR SELENOM TMEM238 IFNG-AS1 CXCR3 AREG

**CD8_Effector**	GZMA, GNLY, GZMK, CTSW	B2M CTSW ZNF683 IL32 CCL5 DUSP1 NKG7 DUSP2 HLA-A AHNAK CST7 CXCR3 MATK LYAR PPP2R5C NCR3 KLRD1 IFITM1 KLRC3 IER2 CD52 SLC9A3R1 FCRL6 IFITM3 EZR HLA-C S100A11 KLF6 IL2RB CD7 SRGN TPST2 ANXA1 MBP IRF1 PIK3R1 HLA-B ZFP36L2 RASSF1 HCST ARID5B FCRL3 PFN1 CAST CD63 FLNA MYO1F TMSB4X ABHD17A ITGB1

Table S1 (Sheet 5). Marker genes and signature genes for NK cell subtypes, related to Fig 1, Fig 2, Fig S1, Fig S2 and Fig S3.		
**Name of NK cell subtypes**	**Canonical genes**	**Signature genes**
**NK_Naive**	SELL, LEF1	LEF1 CCR7 GZMK XCL2 XCL1 MAP3K8 IL2RB CMC1 FOSB MAFF KLRC1 AREG FCER1G IER5 IFITM3 CSRNP1 PMAIP1 RGS1 TENT5C KLRB1 IL18RAP CEBPD ABLIM1 CLDND1 ZFP36 SKIL CXXC5 TRDC MATK GSN CD160 IL12RB2 PIM3 ZBTB16 PRR7 ZNF331 FOSL2 TC2N IFRD1 ICAM1 FAM177A1 PDE4B IER3 HOOK2 HSH2D CAPG SATB1 PPP1R15B
**NK_Memory**	CD27	LEF1 CCR7 MAL CAMK4 RCAN3 CD27 AQP3 SOCS3 PRKCQ-AS1 NCK2 DGKA CD5 IL6ST MYC TRABD2A TESPA1 SNHG7 TRAT1 GPR183 LDLRAP1 PDE3B THEMIS LY9 MAML2 OXNAD1 CD4 BEX3 ABLIM1 AC025164.1 SATB1 PCED1B INPP4B LINC02273 SERINC5 TNFSF8 SUSD3 CD28 EPHX2 ACTN1 TMEM238 ARMH1 CMTM7 BACH2 LINC01550 PAG1 APEX1 NELL2 TC2N AIF1 CNST

**NK_Proliferative**	MKI67	RRM2 STMN1 MCM7 HMGB2 GAPDH MCM5 MCM3 DEK DNMT1 PCNA DUT PPIA IGFBP7 HNRNPAB SLBP CDT1 E2F1 MCM2 DHFR GSTP1 FEN1 GINS2 MCM6 H2AZ2 CLSPN PTMA MCM4 NASP SNRPB RPA3 CENPK RANBP1 SMC1A RALY LMNB1 NUCB2 ATAD2 TMEM106C CFL1 CARHSP1 NUCKS1 USP1 SRSF10 CKS1B TK1 HPRT1 DNAJC9 MZT2A GZMH CD160 GZMB GZMA
**NK_CD56**	CD56(bri), CD160(dim)	KLRC2 B2M LAG3 PTMS KLRC3 CST7 FCGR3A KLRC4 SYNGR1 ADGRG1 TRG-AS1 FCRL6 ZBTB38 PRSS23 KIR2DL3 KIR3DL2 MXRA7 ASCL2 LINC02446 ZNF683 RAP2A LGALS3 F2R NKG7 LILRB1 COL6A2 KIR3DL1 CD320 PTGDS PRSS57 HLA-A CYBA CX3CR1 ABI3 OASL MGST3 RGS9 RAB11FIP5 GNPTAB SLC1A7 SLC2A4RG TTC16 FGL2 LINC01871 TMEM273 GRAP2 SYT11 CASP1 LINC00944 PLAAT3
**NK_CD160**	CD56(dim), CD160(bri)	FCER1G SPON2 CCL4 CLIC3 SH2D1B KLRB1 IGFBP7 CCL3 MYOM2 S1PR5 NKG7 PRF1 CHST2 AKR1C3 CD38 PMAIP1 MAP3K8 CXXC5 CEBPD CX3CR1 IER5 KLRC1 FCGR3A IL2RB ZBTB16 C1orf162 PRSS23 TMIGD2 LINC00299 TTC38 METRNL RHOC TLE1 RIN3 GSN HAVCR2 LAIR2 IGF2R PTPN12 IL18RAP AOAH TXK TBX21 IFITM3 CD160 NFATC2 KLRF1 HSH2D NFKBIB HIPK2

Table S1 (Sheet 6). Marker genes and signature genes for monocytes cell subtypes, related to Fig 1, Fig 2, Fig S1, Fig S2 and Fig S3.		
**Name of monocytes cell subtypes**	**Canonical genes**	**Signature genes**
**Mono_CD14**	CD14	LYZ LGALS2 CD14 VCAN SGK1 MS4A6A IER3 CSF3R GRN AHR AC020656.1 S100A9 TNFAIP2 IL1B CRTAP CPVL PPIF CD36 CDKN1A VIM ALDH2 KLF10 MNDA HLA-DMA NLRP3 CLEC4E NCF1 APLP2 DUSP6 CAPG HLA-DMB CCR1 CCL3L1 FCN1 CCL3 CTSS TREM1 TRIB1 PTPRE SOCS3 CSTA S100A12 MAFB LY86 ZFP36 YBX3 FPR1 IRF2BP2 NAIP FCGR2A
**Mono_CD14_CD16**	CD14 CD16	NRGN PPBP TUBB1 PRKAR2B CAVIN2 GNG11 PF4 CLU SPARC MYL9 MPIG6B F13A1 GP1BB GP9 HIST1H2AC TREML1 ITGA2B VCL ITGB3 MTURN PTGS1 GRAP2 CTSA CMTM5 TPM4 RGS18 PGRMC1 MARCH2 TRIM58 NCOA4 BEX3 LIMS1 PF4V1 ACTN1 AP003068.2 THBS1 MMD MAP3K7CL OAZ1 AP001189.1 ODC1 TSPAN33 MPP1 PDLIM1 C2orf88 SH3BGRL3 TSC22D1 TMEM40 RNF11 YWHAH

**Mono_C1QA**	C1QA/B/C	CD74 HLA-DRA HLA-DQA1 HLA-DRB1 HLA-DMA HLA-DQB1 CST3 HLA-DPA1 HLA-DPB1 TMSB10 HLA-DMB IFI30 CLEC10A PID1 LIPA PEA15 LGALS2 CALHM6 CPVL KLF10 ABI3 CDKN1A CCL3L1 PHPT1 FUOM RHOC C1orf162 NENF ATF5 NPC2 PLEKHO1 GSN SECTM1 CAMK1 NME1 PPP1R14B GPR183 CSF1R NHP2 LGALS3 CTSB DNPH1 LILRB4 HLA-DQA2 VDAC1 HMGA1 SORBS3 LSM4 MARCKSL1 YBX3
**Mono_MDSCs**	S100A8/A9/A12	S100A8 S100A12 S100A9 S100A6 GAPDH VCAN PLBD1 RBP7 TSPO MNDA LYZ CDA PGD CSTA CEBPD S100A4 CD14 VIM RGS2 PADI4 GCA NCF1 CXCL8 CAPG CSF3R RETN BLVRB QPCT MCEMP1 AGTRAP SERF2 CYP1B1 FPR1 HMGB2 MGST1 MEGF9 TKT ATP5F1E VNN2 AC020656.1 TALDO1 MARC1 CD36 CKAP4 BST1 PROK2 HP GLIPR2 MS4A6A H3F3A

Table S2 (Sheet 1). Signature genes usded to define functional status in immune cells			
**Naive scores**	**Exhaustion scores**	**Cytotoxic scores**	**Regulatory effector score**
CCR7, TCF7, LEF1, SELL	HAVCR2,CXCL13,CCL3,SIRPG,IFNG,TIGIT,GZMB,PDCD1,PARK7,TNFRSF9,ACP5,CTLA4,RBPJ,MIR155,CXCR6,CD27,FKBP1A,BST2,TPI1,MIR155HG,PTTG1,CD63,SAMSN1,RGS1,CD27-AS1,ITGAE,MIR4632,HLA-DRA,IGFLR1,KRT86,ENTPD1,DUSP4,SIT1,TOX,PHLDA1,CCND2,GPR25,LAYN,PRDX5,SARDH,FASLG,MIR3917,ANXA5,CTSD,PDIA6,RANBP1,FKBP1A-SDCBP2,COTL1,TNFRSF1B,IDH2,CD38,CD82,LAG3,MIR497HG,APOBEC3C,ITM2A,COX5A,IFI35,NDFIP2,TNFRSF18,KRT81,DNPH1,RGS2,HMGN1,DYNLL1,SNRPB,STRA13,SYNGR2,RAB27A,PSMC3,GALM,FABP5,UBE2L6,MYO7A,PRDX3,DDIT4,STMN1,CDK2AP2,VCAM1,SNAP47,PSMB3,ISG15,HLA-DRB5,CKS2,TNIP3,CD7,PSMD4,ATP6V1C2,PSMD8,HLA-DRB6	PRF1, IFNG, GNLY, NKG7, GZMA, GZMB, GZMH, GZMK, GZMM, KLRK1, KLRB1, KLRD1, FCGR3A, FGFBP2, ZEB2, CTSW, CST7	TNFRSF9,FOXP3,CTLA4,CCR8, ADORA2A, REL, TGFB1,HELIOS

Table S2 (Sheet 2). Inflammatory genes and cytokine score	
**Inflammatory genes**	**Cytokine scores**
ABCA1 ABI1 ACVR1B ACVR2A ADGRE1 ADM ADORA2B ADRM1 AHR APLNR AQP9 ATP2A2 ATP2B1 ATP2C1 AXL BDKRB1 BEST1 BST2 BTG2 C3AR1 C5AR1 CALCRL CCL17 CCL2 CCL20 CCL22 CCL24 CCL5 CCL7 CCR7 CCRL2 CD14 CD40 CD48 CD55 CD69 CD70 CD82 CDKN1A CHST2 CLEC5A CMKLR1 CSF1 CSF3 CSF3R CX3CL1 CXCL10 CXCL11 CXCL6 CXCL8 CXCL9 CXCR6 CYBB DCBLD2 EBI3 EDN1 EIF2AK2 EMP3 EREG F3 FFAR2 FPR1 FZD5 GABBR1 GCH1 GNA15 GNAI3 GP1BA GPC3 GPR132 GPR183 HAS2 HBEGF HIF1A HPN HRH1 ICAM1 ICAM4 ICOSLG IFITM1 IFNAR1 IFNGR2 IL10 IL10RA IL12B IL15 IL15RA IL18 IL18R1 IL18RAP IL1A IL1B IL1R1 IL2RB IL4R IL6 IL7R INHBA IRAK2 IRF1 IRF7 ITGA5 ITGB3 ITGB8 KCNA3 KCNJ2 KCNMB2 KIF1B KLF6 LAMP3 LCK LCP2 LDLR LIF LPAR1 LTALY6E LYN MARCO MEFV MEP1A MET MMP14 MSR1 MXD1 MYC NAMPT NDP NFKB1 NFKBIA NLRP3 NMI NMUR1 NOD2 NPFFR2 OLR1 OPRK1 OSM OSMR P2RX4 P2RX7 P2RY2 PCDH7 PDE4B PDPN PIK3R5 PLAUR PROK2 PSEN1 PTAFR PTGER2 PTGER4 PTGIR PTPRE PVR RAF1 RASGRP1 RELA RGS1 RGS16 RHOG RIPK2 RNF144B ROS1 RTP4 SCARF1 SCN1B SELE SELENOS SELL	IL2 IL7 CSF3 CXCL10 CCL2 CCL3TNF IL6 CCL7 IL1RN CSF1 IFNG IL2RA IL10 IL18 HGF CXCL9 CCL27 TGFB1 IL1B LTA CSF2 LTB TNFSF13 IL4 CCL12 CXCL8 CXCL11 CCL4 CXCL1 CXCL2 CXCL3 CCL3L1 CCL8 CXCL16 IFNA1 CCL5 CCL11 IFNA2 CCL20 CCL4L2 OSM TNFSF14 SA100A12 FGF19 CXCL5 CCL19 IL18R1 TGFA IFNB1 IL8 IL17C TNFSF10 FGF7 XCL1 FGF13 LIF TGFB3 INHBE CERS1 TXLNA IFNW1 IL22 XCL2 CCL25 CCL16 CD40LG IL20 FASLG TPO SCYL3 PF4V1 TNFSF8 GDF15 IL1A VEGFA GDF7 BMP6 PDGFA IL21 ABCD-1 ABCD-2 PDGFB TNFSF4 FAM19A1 HBEGF PDGFD IL12RB2 GH1 VEGFB MIP3B IL27 PF4 BMP8B TNFSF12 IL15 SCYL2 SCYL1 TSLP GDF11 SDF1B INHBA PPBP FGF11 IFNG-AS1 FGF22 VEGFC CCL18 TNFSF11 IL12A

SEMA4D SERPINE1 SGMS2 SLAMF1 SLC11A2 SLC1A2 SLC28A2 SLC31A1 SLC31A2 SLC4A4 SLC7A1 SLC7A2 SPHK1 SRI STAB1 TACR1 TACR3 TAPBP TIMP1 TLR1 TLR2 TLR3 TNFAIP6 TNFSF14 TNFRSF1B TNFRSF9 TNFSF10 TNFSF15 TNFSF9 TPBG VIP	EBI3 AMH IL26 IL32 PDGFC FGF23 IGF1 IL1F11 CCL28 CLCF1 TNFSF9 BMP3 IL24 GDF10 CXCL6 GDF9 IL23A IL16 CD70 IL5 FGF9 IFNL1 TSC1 FGF2 IL23R IL1G SPP1 IL12RB1 BMP4 IL13 TPAR1 TGFB2 FAM19A2 AGIF3 EDA MIF TNFSF13B BMP7 FGF18 CCL23

Table S2 (Sheet 3). Signature genes related to IFN-response		
**Response To Interferon Alpha (GO:0035455)**	**Response To Interferon Beta (GO:0035456)**	**RESPONSE_TO_TYPE_I_INTERFERON（GO:0034340）**
ADAR AXL BST2 EIF2AK2 GAS6 GATA3 IFITM1 IFITM2 IFITM3 IFNAR1 IFNAR2 KLHL20 LAMP3 MX2 PDE12 PYHIN1 RO60 TPR	ACOD1 AIM2 BST2 CAMK2A CAPN2 CDC34 HTRA2 IFI16 IFITM1 IFITM2 IFITM3 IFNAR2 IFNB1 IKBKE IRF1 IRGM MNDA NDUFA13 OAS1 PLSCR1 PNPT1 PYDC5 PYHIN1 SHFL STAT1 STING1 TLR3 TREX1 TRIM6 UBE2G2 UBE2K XAF1	ADAR AZI2 CACTIN CDC37 CH25H CNOT7 DCST1 EIF4E2 FADD GIGYF2 HDAC4 IFI27 IFIH1 IFIT1 IFITM1 IFITM2 IFITM3 IFNA1 IFNA10 IFNA13 IFNA14 IFNA16 IFNA17 IFNA2 IFNA21 IFNA4 IFNA5 IFNA6 IFNA7 IFNA8 IFNAR1 IFNAR2 IFNB1 IFNE IFNK IFNW1 IKBKE IRAK1 IRF3 IRF7 ISG15 JAK1 LSM14A MAVS METTL3 MIR21 MMP12 MUL1 MX1 MYD88 NLRC5 OAS1 OAS2 OAS3 PTPN1 PTPN11 PTPN2 PTPN6 RBM47 RNF185 SAMHD1 SETD2 SHFL SHMT2 SIN3A SMPD1 SP100 STAT1 STAT2 STING1 TANK TBK1 TBKBP1 TRAF3 TREX1 TRIM41 TRIM56 TRIM6 TRIM65 TTLL12 TYK2 UBE2K USP18 USP27X USP29 WNT5A YTHDF2 YTHDF3 ZBP1

Table S2 (Sheet 4). Platelet activation and platelet aggregation	
**Platelet activation (GO:0030168）**	**Platelet aggregation（GO:0070527）**
ACTB ACTG1 ACTN1 ADAMTS13 ADAMTS18 ADRA2A ADRA2B ADRA2C ALOX12 APOE AXL BLOC1S3 BLOC1S4 C1GALT1C1 C1QTNF1 CD40 CD40LG CD9 CEACAM1 CELA2A CLIC1 COL3A1 COMP CSRP1 CTSG DGKA DGKB DGKD DGKE DGKG DGKH DGKI DGKK DGKQ DGKZ EMILIN1 EMILIN2 ENTPD2 F11R F2 F2R F2RL2 F2RL3 FCER1G FERMT3 FGA FGB FGG FGL1 FIBP FLNA FUNDC2 FZD6 GATA1 GNA13 GNAQ GNAS GP1BA GP1BB GP5 GP6 GP9 HBB HRG HSPB1 IL6 IL6ST ILK ITGB3 JAK2 LCK LYN MAPK14 MERTK METAP1 MFSD2B MMRN1 MYH9 MYL12A MYL9 NOS3 P2RX1 P2RY1 P2RY12 PDGFA PDGFB PDGFRA PDIA2 PDIA3 PDPN PEAR1 PF4 PIK3CA PIK3CB PIK3CG PLA2G4A PLCG2 PLEK PLSCR1 PPIA PRKCA PRKCD PRKCQ PRKG1 PTPN6 RAP2B SAA1 SELP SERPINE2 SH2B3 SLC6A4 SLC7A11 SRC SRF STXBP1 STXBP3 SYK TEC THBD TLN1 TLR4 TREML1 TSPAN32 TUBB1 TXK TYRO3 UBASH3B VAV1 VAV2 VAV3 VCL VPS33B VWF WNT3A	ACTB ACTG1 ACTN1 ADAMTS18 ALOX12 BLOC1S4 C1QTNF1 CD9 CEACAM1 CELA2A CLIC1 COMP CSRP1 CTSG EMILIN1 EMILIN2 F11R F2RL3 FERMT3 FGA FGB FGG FGL1 FIBP FLNA GATA1 GNAS GP6 HBB HSPB1 IL6 IL6ST ILK ITGB3 JAK2 LYN METAP1 MFSD2B MMRN1 MYH9 MYL12A MYL9 P2RY12 PDGFRA PDIA2 PDIA3 PDPN PEAR1 PIK3CB PIK3CG PLEK PPIA PRKCA PRKCD PRKCQ PRKG1 PTPN6 RAP2B SERPINE2 SH2B3 SLC6A4 SLC7A11 STXBP1 STXBP3 SYK TLN1 TSPAN32 TUBB1 TYRO3 UBASH3B VCL VPS33B WNT3A

Table S3. The clinical features and laboratory findings of enrolled KD patients.

		IVIG responsive KD patients					IVIG non-responsive KD patients						
		P1	P2	P3	P4	P5	P1	P2	P3	P4	P5	P6	P7
Signs and symptoms	Fever (>37.3 °C)	Yes	Yes	Yes	Yes	Yes	Yes	Yes	Yes	Yes	Yes	Yes	Yes
	Rash	No	Yes	Yes	Yes	Yes	No	Yes	Yes	Yes	No	Yes	Yes
	ErYesthema and cracking of lips, strawberrYes tongue	Yes	Yes	Yes	Yes	Yes	Yes	Yes	No	Yes	Yes	Yes	Yes
	ErYesthema and edema of the hands and feet	Yes	Yes	Yes	Yes	Yes	Yes	No	Yes	Yes	Yes	Yes	Yes
	Bulbar conjunctival injection	Yes	Yes	Yes	Yes	Yes	Yes	Yes	Yes	Yes	Yes	No	No
	Cervical lYesmphadeNopathYes (≥Yes.5 cm diameter)	Yes	Yes	Yes	Yes	Yes	Yes	Yes	Yes	Yes	Yes	Yes	Yes
Laboratory fndings	Whiteblood cell count$\times 10^9$ (3.97-9.15$\times 10^9$/L)	9.53	18.41	13.16	12.07	21.25	31.71	11.48	20.06	7.99	7.16	12.39	11.84
	Percent of neutrophil count (%)	51.3	57.2	74	65.7	56.9	83	62.7	65.1	71.8	52	77.1	73.3
	Percent of lymphocyte (%)	38	35.7	20.3	21.8	29.5	12	34.8	24.8	21.2	37	13.8	22.9
	Hemoglobin (g/L)	107	104	116	115	92	124	101	106	120	109	118	109
	Platelet count ×109/L	312	376	442	340	413	434	259	533	176	238	254	406
	CRP (0-5.0mg/L)	31.3	61.53	24.99	156	105	24.1	99	180	72.03	47	75	49
	Coronary artery dilatation	No	Yes	No	No	No	No	No	No	No	No	No	No

Software and algorithms

Software	Source	Website
anadata	pypi	https://github.com/theislab/anndata
CellRanger v3.x	10x Genomics	http://10xgenomics.com
ggplot	bioconductor	https://ggplot2.tidyverse.org
ggpubr	bioconductor	https://github.com/kassambara/ggpubr
gseapy-0.10.7	pypi	https://pypi.org/project/gseapy
harmonypy	pypi	https://github.com/slowkow/harmonypy
kallistobustools	pypi	https://github.com/pachterlab/kb_python
scanpy v1.9.2	bioconda	https://github.com/theislab/scanpy
scrublet v0.7.6	bioconda	https://github.com/icbi-lab/scirpy
scrublet v0.2.3	pypi	https://github.com/swolock/scrublet
statannot	pypi	https://pypi.org/project/statannot
